# Geochemical signatures in plastic debris from the Curonian Lagoon, Lithuania

**DOI:** 10.1371/journal.pone.0340582

**Published:** 2026-02-02

**Authors:** Sajjad Abbasi, Neda Hashemi, Patryk Oleszczuk, Viktorija Sabaliauskaitė, Nerijus Dzingelevičius, Arūnas Balčiūnas, Rita Vaičekauskaitė, Reda Dzingelevičienė

**Affiliations:** 1 Department of Earth Sciences, College of Science, Shiraz University, Shiraz, Iran; 2 Marine Research Institute, Klaipeda University, Klaipeda, Lithuania; 3 Géosciences Environnement Toulouse, CNRS, IRD, Université de Toulouse, Toulouse, France; 4 Department of Radiochemistry and Environmental Chemistry, Faculty of Chemistry 3 Maria Curie-Skłodowska Square, Lublin, Poland; 5 Klaipėdos Valstybinė Kolegija/Higher Education Institution, Klaipeda, Lithuania; 6 Faculty of Health Sciences, Klaipeda University, Klaipeda, Lithuania; Amity University Amity Institute of Biotechnology, INDIA

## Abstract

This study examined elemental accumulation on weathered plastic waste as a contributor to environmental pollution. Twenty-five plastic samples collected near the Curonian Lagoon in Lithuania were analyzed for 32 elements using inductively coupled plasma-mass spectrometer (ICP-MS). Five common polymers (polyethylene, polypropylene, Polyethersulfone, polyester, and polyethylene terephthalate) were identified, with polyethylene exhibiting the highest elemental uptake, followed by polypropylene and polystyrene. Correlation analysis suggested relationships between elemental uptake and geochemical behavior, with alkali and alkaline earth elements (REEs) potentially enhancing the uptake of intermediate ions. However, elements such as sulfur, lead, cadmium, and antimony showed limited correlation with other elements. Despite their low mobility, REEs were used to infer sources of pollution, and the aluminum to lanthanum ratio was proposed as a potential indicator of possible anthropogenic pollution from industrial, petroleum, and vehicle emissions.

## Introduction

Plastics have become indispensable to modern society since their inception in the early 20th century, with global production soaring to over 400 million tons as of 2024 [1]. However, the durability that makes these materials useful also contributes to a pervasive environmental crisis, as mismanaged plastic waste increasingly contaminates aquatic ecosystems [2–5]. Common polymers in aquatic environments like polyethylene, polypropylene and polystyrene, are not merely physical pollutants; they are chemically active particles that contain hazardous additives and, once in the environment, act as vectors for a wide range of pollutants [6–9].

The role of plastics as pollutant vectors is twofold. First, they can release inherent chemical ingredients, including organic additives and, as recently identified, rare earth elements (REEs) which have been proposed as tracers for electronic waste in plastics [10–12]. Second, and perhaps more significantly, their surfaces efficiently sorb exogenous contaminants from the surrounding environment. These include toxic elements [9] and persistent organic pollutants such as PCBs, PAHs, and antibiotics [13]. This sorptive capacity is dramatically enhanced by environmental aging and weathering, which increase the surface area and reactivity of plastic debris [14–16]. The adsorption processes are governed by various mechanisms, including hydrophobic interactions, van der Waals forces, and pore filling, dictated by the physicochemical properties of both the polymer and the contaminant [17,18].

The risk posed by plastic adsorbed contaminants is particularly acute in sensitive and enclosed aquatic systems, such as coastal lagoons and wetlands. These ecosystems, often separated from the open sea by spits or barrier islands, are highly susceptible to pollution accumulation from diverse anthropogenic sources, industrial effluents and oil pollution, vehicle emissions, agricultural activities, and tourism [2,9,12,19–22]. In confined systems, the interaction of pollutants with plastic particles can extend the residence time and bioavailability of toxins, increasing their potential to enter the food web and magnify ecological risks [6].

The Curonian Lagoon, the largest coastal lagoon in Europe, represents a critical case study of such pressures. As a protected area of high ecological and economic value [23], it faces significant pollution inputs from oil activities in its northern section, agricultural practices in its watershed, and tourism [19,24]. Existing research has documented both plastic pollution and elemental contamination in the region. For instance, atmospheric deposition has been identified as a major pathway for Microplastics influx (27.8 × 10^12^ MP year ⁻ ¹), far exceeding riverine input (20.7 × 10^9^ MP year ⁻ ^1^) [19], with high concentrations (60.3 MP L^-1^) also found in ice cores [25]. Older studies have been conducted on the evaluation of toxic elements. Prior research indicates significant anthropogenic enrichment of toxic elements (Cd, Cu, Pb, As) in lagoon sediments, mainly from the northern part and Klaipėda port [26–28].

While research on elemental adsorption onto plastics is expanding in both field [15,19,29,30] and laboratory settings [6,31–33], a critical gap remains in understanding the accessible fraction of these adsorbed elements, the portion that is potentially bioavailable. Traditional methods involving strong acid digestion of the entire plastic matrix can introduce contamination and overestimate risk by measuring elements locked within the polymer itself. Therefore, this study examines the bioavailable fraction and potential risks of elements adsorbed onto plastic debris from the Curonian Lagoon. We used ICP-MS with a modified BCR sequential extraction (0.11 M acetic acid [34]), previously applied in our study [6], to analyze the bioavailable elemental phase. This approach enables a more environmentally relevant analysis of polymer adsorption behavior. Additionally, the study employs a geochemical approach to explore potential contamination sources using REE signatures on plastic surfaces.

## Materials and methods

### Sample collection and polymer identification

Twenty-five deteriorated plastic pieces, identified by physical damage, discoloration, or adhered sediment, were collected from the Curonian Lagoon, Lithuania, on September 18, 2024. Sampling was conducted within a 100–200 m radius of sites previously studied by Abbasi et al. [19], with permission granted by Klaipėda University. Items ranged from 1 to 6 cm and were transported in glass containers. Loosely adhered debris were removed by gentle agitation in deionized water for 72 h, after which samples were air dried at room temperature for 24 h. Polymer type was determined by visual inspection of resin codes and ATR FTIR spectroscopy. ATR FTIR measurements were performed on a Nicolet 8700A spectrometer (diamond ATR) with 64 scans, 4000–400 cm ⁻ ¹, 4 cm ⁻ ¹ resolution. Spectra were matched against reference spectra in the OpenSpecy database (https://openanalysis.org/openspecy/Spectra). The five most prevalent polymers were polyethylene (PE), polypropylene (PP), polystyrene (PS), Polyethersulfone (PES) and polyethylene terephthalate (PET). Each item was ground to a homogenate using a pestle and mortar, stainless steel scissors, and a screw driven grinder, then sieved through a 2 mm stainless steel mesh. The resulting powder was stored in glass vials.

### Extraction and digestion

To extract exchangeable metals from the polymer surface and associated biofilm, we used the first step of a modified BCR sequential extraction protocol [34] with 0.11 M acetic acid -. This is a new approach to measuring the available (not residual) concentration of elements, which was first performed in a previous study to investigate the concentration of exchangeable elements bound to polymers [6]. Thus, we followed the protocol of the previous study to ensure that the results were comparable. Initially, 20 ml of 0.11 M acetic acid (pH ≈ 3) was added to each sample tube. The tubes were then shaken for 16 h at room temperature on a double-head shaker at 30 rpm. Following shaking, the samples were centrifuged at 400 rpm for 20 min. The resulting supernatant was carefully decanted into 60 ml Teflon centrifuge tubes, ensuring no plastic material was transferred. The remaining solid residue was rinsed with 20 mL of deionized water for 15 minutes, and the rinses were discarded.

### Quality assurance

Quality assurance measures included the use of 3 types of blanks to monitor potential contamination during analysis. Procedural blank, encompassing all reagents and containers, were processed without the introduction of plastic materials. Sieve blank was prepared by rinsing a clean 2 mm stainless steel sieve and treating it in the same manner as the samples. Polymer blank, utilizing a clean polymer, were also processed identically. To assess the accuracy of the extraction protocol, the agricultural soil certified reference material (CRM) NCS ZC71009 (China National Analysis Center) was digested in triplicate.

### Instrumental analysis

Elemental analysis was performed on 25 fractionated composite samples (13 PE, 4 PP, 3 PS, 4 PES, and 1 PET), 13 duplicates, and three control samples (processed identically but without plastic material). Elemental analysis, including Group 1 and 2, transition elements, and REEs, was performed using inductively coupled plasma-mass spectrometer (ICP-MS) on a Thermo Scientific iCAP 7400 Duo. Argon served as the plasma generator gas (flow rate 12 L min^−1^) and nebulization system gas (flow rate 0.5 L min^−1^). External standards (0.01 to 5 mg L^−1^) were prepared by diluting a Merck multi-standard solution (HC87632955) in 2% HNO_3_. Procedural blanks, processed with each sample batch, showed low and consistent values. The Method Detection Limit (MDL), as provided by the analytical laboratory and derived from reagent blanks, ranged from 0.1 μg L ⁻ ¹ to 1 μg L ⁻ ¹ for REEs and 1 μg L ⁻ ¹ to 100 μg L ⁻ ¹ for other elements. The corresponding Method Quantification Limit (MQL), calculated as 3.3 times the MDL [35], ranged from 0.33 μg L ⁻ ¹ to 3.3 μg L ⁻ ¹ for REEs and 3.3 μg L ⁻ ¹ to 330 μg L ⁻ ¹ for other elements. These were converted to mass-normalized units for direct comparison with sample data (reported in mg kg^-1^, except for Li, Cd, Sb, and REEs, which are reported in μg kg^-1^, Table 1), and all sample concentrations were blank-subtracted and well above the MQL. The accuracy of the extraction and analysis was verified using a certified reference soil (e.g., NIST 2711a), a justified surrogate for the challenging environmental matrices associated with weathered plastics, with recoveries ranging from ~70% for Cu and Pb to >90% for other elements. Repeatability, expressed as relative standard deviation, was better than 10% for all elements.

**Table 1:** Detection limits (MDL; µg L^-1^) of elements, normalized to plastic weight and solution volume (mg kg^-1^) expect for Li, Cd, Sb, and REEs (µg kg^-1^).

Elements	MDL (µg L^-1^)	MDL (µg kg^-1^)	Elements	MDL (µg L^-1^)	MDL (µg kg^-1^)
Ag	1	0.03	Li	1	27.65
Al	100	0.29	Cd	1	27.15
As	1	0.03	Sb	1	25.36
Ba	1	0.03	Ce	1	28.15
Ca	100	0.29	Dy	0.1	2.82
Co	1	0.03	Eu	0.1	2.51
Cr	1	0.03	Gd	0.5	13.29
Cu	100	0.29	La	1	28.16
Fe	100	0.29	Lu	0.1	2.63
K	100	0.29	Nd	0.5	14.68
Mg	100	0.29	Sm	1	25.34
Mn	100	0.29	Y	1	28.15
Na	100	0.29			
Ni	100	0.28			
P	100	0.29			
Pb	1	0.03			
Rb	1	0.03			
S	100	0.29			
Si	100	0.29			
Sr	100	0.29			
Zn	100	0.29			

### Data handling and statistics

Statistical analyses were performed using Excel 365 and SPSS 27. The Shapiro-Wilk test was used to assess normality. Depending on the distribution and sample sizes, Kruskal-Wallis tests were conducted, followed by post-hoc pairwise comparisons using the Dwass-Steel-Critchlow-Fligner method. The False Discovery Rate (FDR) was controlled for all multiple comparisons, and results are reported with adjusted q-values, along with the number of samples used for each test. Correlation analyses included robust measures (Spearman) with 95% confidence intervals where possible. For groups with n = 1, no inferential statistics were performed; results are presented descriptively.

## Results

### Element concentrations overview

Elemental analysis of plastic samples revealed varying abundance and distribution patterns. The abundance of elements decreased in the order: Ca (875.96 ± 474.17 mg kg^-1^)> Na (175.43 mg kg^-1^) > S (167.32 ± 73.75 mg kg^-1^)> Mg (162.53 ± 109.12 mg kg^-1^)> Mn (40.96 ± 39.36 mg kg^-1^) > K (40.01 ± 24.22 mg kg^-1^)> Si (30.64 ± 18.45 mg kg^-1^) > P (26.97 ± 21.87 mg kg^-1^)> Al (14.55 ± 6.93 mg kg^-1^)> Sr (11.88 ± 7.94 mg kg^-1^)> Ba (6.02 ± 3.79 mg kg^-1^)> Pb (1.47 ± 1.67 mg kg^-1^)> Cu (1.15 ± 0.68 mg kg^-1^)> Cr (0.9 ± 0.95 mg kg^-1^)> Ni (0.44 ± 0.15 mg kg^-1^)> As (0.3 ± 0.13 mg kg^-1^)> Rb (0.19 ± 0.09 mg kg^-1^)> Co (0.14 ± 0.09 mg kg^-1^)> Ag (0.11 ± 0.13 mg kg^-1^)> Ce (95.16 ± 24.22 µg kg^-1^)> Cd (79.61 ± 21.17 µg kg^-1^)> Li (76.27 ± 29.52 µg kg^-1^) > Y (61.46 ± 16.03 µg kg^-1^)> La (55.24 ± 12.41 µg kg^-1^)> Nd (44.74 ± 21.72 µg kg^-1^)> Sb (29.32 ± 12.55 µg kg^-1^)> Sm (30.81 ± 1.45 µg kg^-1^)> Gd (19.35 ± 2.9 µg kg^-1^)> Dy (7.62 ± 2.19 µg kg^-1^)> Lu (2.70 ± 0.19 µg kg^-1^)> Eu (4.09 ± 0.22 µg kg^-1^). In groups one and two, all alkali and alkaline earth elements were detected in 100% of samples except for Li (12%). Among intermediate elements, Ag, Co, and Sb were detected in 96%, 84%, and 76% of samples, respectively, while Cd and Ni were found in 68%, Cr in 24%, and As in only 8%. All other intermediate elements were present in 100% of the samples. For REEs, Nd was present in 96% of the samples, Y and Ce in 72%, La and Dy in 68%, Gd in 56%, Eu in 28%, Sm in 25%, and Lu in 12%.

Kruskal-Wallis tests revealed significant differences in adsorbed concentrations among polymer types for Dy, La, Y, Na, Cu, K, P, S, and Sr (S1 Table, p < 0.05). Post-hoc pairwise comparisons using the Dwass-Steel-Critchlow-Fligner method, with FDR control, indicated the most significant difference prior to q-value adjustment was between PES-PE PS-PE and PS-PP (p < 0.05). However, after FDR correction, only Cu and P exhibited significant differences between PP and PS (q < 0.05, rank difference = 1), while Na and S showed the largest difference between PES and PE (q < 0.05, rank difference = 1). K, Sr, Y, La, and Dy showed no significant differences between any polymer pairs after FDR control (all q > 0.05), warranting cautious interpretation.

**Fig 1:**
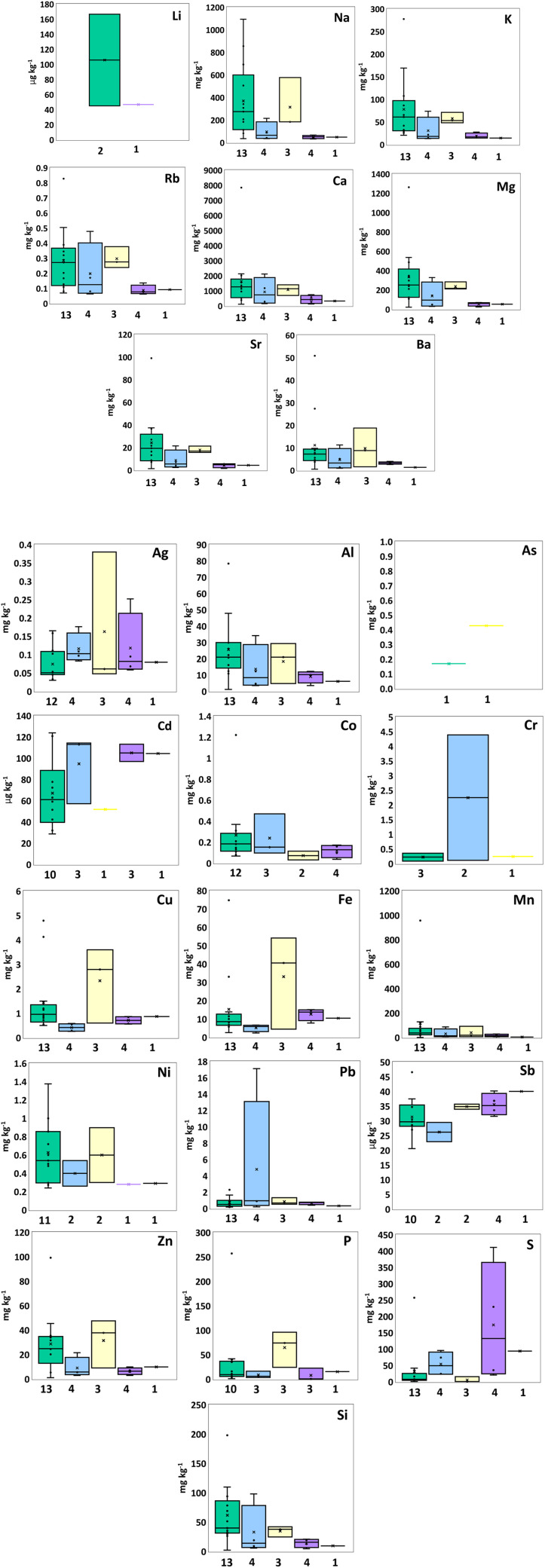
Box plots summarize intermediate elements concentrations in PE (green), PP (blue), PS (yellow), PES (purple), and PET (black), displaying median, mean (×), interquartile range, minimum, maximum, and outliers.

**Fig 2:**
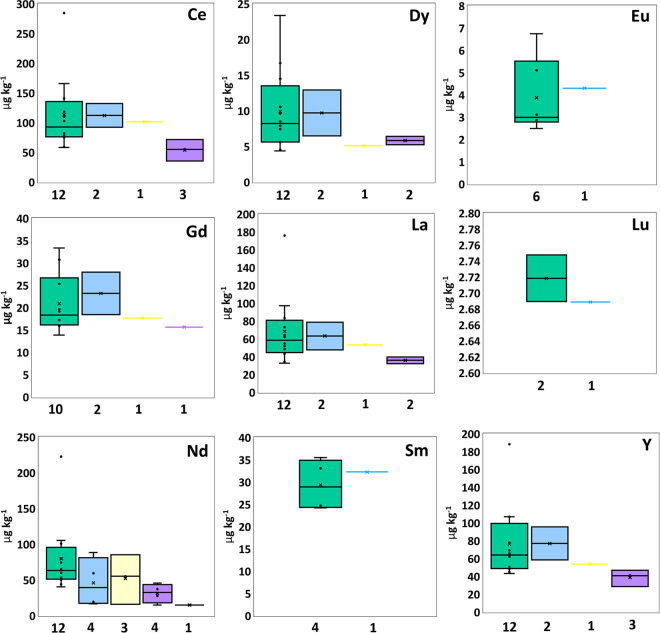
Box plots summarize rare earth element (REEs) concentrations in PE (green), PP (blue), PS (yellow), PES (purple), and PET (black), displaying median, mean (×), interquartile range, minimum, maximum, and outliers.

The highest mean abundances for all group one and two elements, including Li (105.78 ± 60.82 µg kg^-1^), Na (369.83 ± 313.97 mg kg^-1^), K (78.05 ± 69.88 mg kg), Ca (1630.50 ± 1886.78 mg kg^-1^), Mg (333.60 ± 303.16 mg kg^-1^), Sr (24.26 ± 24.26 mg kg^-1^) and Ba (11.15 ± 13.01 mg kg^-1^), were found in PE, except for Rb which was highest in PS (0.3 ± 0.06 mg kg^-1^) (Fig 1). For intermediate elements, PE exhibited the highest average abundance of Al (25.63 ± 18.68 mg kg^-1^), Co (0.27 ± 0.30 mg kg^-1^), Mn (115.77 ± 244.93 mg kg^-1^), Ni (0.62 ± 0.33 mg kg^-1^), S (258.80 ± 66.15 mg kg^-1^), and Si (61.74 ± 48.69 mg kg^-1^). PP contained the highest average concentrations of Cr (2.25 ± 2.14 mg kg^-1^) and Pb (4.80 ± 7.12 mg kg^-1^). PS showed the highest levels of Ag (0.16 ± 0.15 mg kg^-1^), As (0.43 mg kg^-1^), Cu (2.33 ± 1.27 mg kg^-1^), Fe (33.07 ± 20.96 mg kg^-1^), Zn (31.44 ± 16.38 mg kg^-1^), and P (64.62 ± 30.07 mg kg^-1^). Cd was highest in PES (104.8 ± 6.59 µg kg^-1^), while Sb was highest in PET (40.1 µg kg^-1^ from one sample) (Fig 1). Regarding REEs, PE had the highest mean concentrations of Ce (113.14 ± 60.21 µg kg^-1^), Dy (9.89 ± 5.45 µg kg^-1^), La (68.46 ± 37.19 µg kg^-1^), Nd (79.73 ± 47.39 µg kg^-1^) and Lu (2.72 ± 0.03 µg kg^-1^). PP exhibited the highest mean concentrations of Gd (23.19 ± 4.77 µg kg^-1^), Y (76.92 ± 18.53 µg kg^-1^) and Sm and Eu from one sample (32.26 and 4.30 µg kg^-1^) (Fig 2). Outliers (very high concentrations) were observed for all elemental groups in PE.

### Correlation analysis

**Fig 3:**
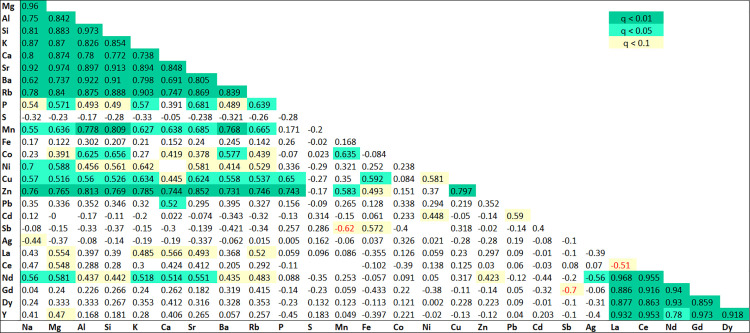
The correlation matrix of element concentrations highlights significant pairwise relationships (abundance > 50%). Significance levels: q < 0.01, dark green; q < 0.05, light green; q < 0.1, yellow; negative correlations, red.

Spearman correlation analysis identified significant pairwise relationships among elements with >50% abundance adsorbed onto plastic polymers (S2 Table). Following False Discovery Rate (FDR) correction (q < 0.01, dark green; q < 0.05, light green), distinct geochemical groups emerged, with correlation precision quantified by 95% confidence intervals. Correlations that lost significance after FDR correction (p < 0.05, q < 0.1) are highlighted in yellow in Fig 3.

A very strong and precisely-estimated positive correlation was observed within a group of alkali and alkaline earth metals. Key pairs, such as Mg–Sr (ρ = 0.974, 95% CI [0.939, 0.989]) and Na–Mg (ρ = 0.958, 95% CI [0.905, 0.982]), demonstrate that the true relationship is not only strong but also reliably estimated to be near the upper limit of the correlation scale. This group was further characterized by its strong positive correlations with the crustal elements Si and Al (e.g., Na–Si ρ = 0.806, 95% CI [0.595, 0.913]).

REEs, including La, Ce, Nd, Gd, Dy, and Y, formed an exceptionally cohesive and reliably-defined cluster. They exhibited near-perfect positive correlations among themselves, as seen in pairs like Ce–La (ρ = 0.971, 95% CI [0.916, 0.990]) and Gd–Y (ρ = 0.973, 95% CI [0.905, 0.992]), indicating a single, well-mixed source.

Among the trace metals, Cu and Zn showed a very strong positive correlation (ρ = 0.797, 95% CI [0.578, 0.909]). While this indicates a robust relationship, the wider confidence interval compared to the REE or crustal groups suggests a greater degree of uncertainty in the exact strength of this association. Furthermore, these elements were significantly correlated with the crustal elements Al and Si (e.g., Al–Zn ρ = 0.813, 95% CI [0.608, 0.916]).

In contrast, Sb demonstrated a significant negative correlation with Mn (ρ = −0.619, 95% CI [−0.842, −0.216]). The fact that the entire confidence interval is below zero and covers a large negative range reinforces the certainty of this inverse relationship. Ag showed weak to moderate negative correlations with Ce and Nd (ρ = −0.507 and −0.555, respectively); however, the associated uncertainty was considerable, with the CI for Ag-Ce including values very close to zero (95% CI [−0.800, −0.020]). Elements such as S and Pb showed few significant correlations with the major geochemical groups, and the relationships that were detected often had wide confidence intervals, indicating uncertain and potentially weak associations.

### Comparison with previous studies

The concentrations of elements associated with plastics in this study were compared with findings from previous research (Table 2). Using the same acetic acid digestion method, a study conducted in Lublin, Poland [6], showed generally lower average concentrations of most elements compared to the Lithuanian samples, with the exception of Cd. Cd concentrations were approximately five times higher in the Polish study, although Cd was detected in a larger number of samples in Lithuania (16) compared to Poland (4). Ni concentrations were comparable between the two locations. Unlike the Polish study [6], which sampled both natural and industrial areas, this study focused solely on the Curonian Lagoon. Higher toxic element concentrations than those reported in the Polish study suggest potential contamination of the Lagoon.

**Table 2:** Comparison of the concentrations of the studied elements with other previous studies.

Concentration (mg kg^-1^)												Method	Environment	Studies
As	Co	Cr	Cu	Fe	Mn	Ni	Pb	Zn	Ca	Mg	Ba			
0.17	0.27	0.22	1.44	15.27	115.77	0.62	0.73	28.70	1630.50	333.60	11.15	acetic acid	Curonian Lagoon, Lithuania	This Study
ND	0.24	2.25	0.42	5.18	30.08	0.40	4.80	8.97	929.47	137.12	4.66			
0.43	0.07	0.24	2.33	33.07	39.53	0.60	0.84	31.44	1074.73	236.60	9.73			
ND	0.12	ND	0.71	12.57	15.95	0.28	0.66	6.38	425.43	53.24	3.23			
ND	0.03	ND	0.87	10.42	3.47	0.29	0.32	9.84	319.65	52.12	1.34			
0.30	0.14	0.90	1.15	15.30	40.96	0.44	1.47	17.07	875.96	162.53	6.02			
	0.03	0.02	0.07	2.78	7.58	0.24	0.03	3.63				acetic acid	Various environment of Lublin Province, Poland	Abbasi et al. [6]
	0.17	0.02	0.45	5.39	39.46	0.43	0.31	18.30						
	0.08	0.03	0.35	3.75	31.85	0.06	0.09							
	0.09	0.03	0.29	3.97	26.30	0.25	0.15	10.97						
0.13		0.18	1.11		1.04	1.15	0.59	8.70			19.11	HNO_3_ + HCL	Australian coastline	Carbery et al. [38]
			3.20	202.25			0.86	21.16				HNO_3_ + 30% HCL	The coast of Bahia, Brazil	Souza et al. [37]
	0.02	0.37	0.73	63.20	6.17	0.09	1.04	11.82				HNO_3_ + 10% HCL	The beaches of south west England	Holmes et al. [15]
	0.14	0.43	0.42	34.40	0.71	0.30	0.11	0.20				0.03 M HCl	laboratory conditions	Turner &Holmes [36]
									50000	5000		70% HNO_3_	Australian coastline	Lee et al. [29]
**Concentration (µg kg**^**-1**^)												**Method**	**Environment**	**Studies**
**Cd**	**Ce**	**Dy**	**Eu**	**Gd**	**La**	**Lu**	**Nd**	**Sm**	**Y**					
67.10	113.14	9.89	3.87	20.89	68.46	2.72	79.73	29.36	76.23			acetic acid	Curonian Lagoon, Lithuania	This Study
94.74	112.07	9.70	4.30	23.19	63.26	2.69	45.88	32.26	76.92					
ND	101.50	5.09	ND	17.66	53.29	ND	52.07	ND	53.89					
104.80	53.95	5.82	ND	15.64	35.92	ND	31.28	ND	38.79					
ND	ND	ND	ND	ND	ND	ND	14.77	ND	ND					
88.88	95.16	7.62	4.09	19.35	55.24	2.70	44.74	30.81	61.46					
9.01												acetic acid	Various environment of Lublin Province, Poland	Abbasi et al. [6]
822.80														
ND														
415.90														
210.53													Australian coastline	Carbery et al. [38]
440	371.00	16.86	6.34	24.40	200.50	1.21	159.35	24.64	101.70			HNO_3_ + 30% HCL	The coast of Bahia, Brazil	Souza et al. [37]
38.46												HNO_3_+ HCL	The beaches of south west England	Holmes et al. [15]
5.00	540	120	99	120	880	8	380	160	340			HNO_3_ + 30% HCL	laboratory conditions	Turner et al. [11]

When comparing our results with studies employing nitric and hydrochloric acid digestion methods in laboratory conditions [36] and studies of natural environments [15,37,29,38], several trends emerged. Given the lack of research on elemental adsorption to plastics in wetland environments, coastal environments were considered the most relevant natural analogue. According to overall abundance, our concentrations are broadly in the same order of magnitude as coastal studies, but direct quantitative comparisons are limited by extraction schemes. Even with weaker acid digestion, Cd, Cu, Co, Cr, Zn, Mn, and Pb concentrations surpassed those of previous studies, an increase not seen for Ba, Ca, or Fe. This suggests that enclosed wetlands may be more vulnerable to toxic metal contamination than coastlines.

## Discussion

### Polymer properties and environmental controls on elemental adsorption

This study supports existing research indicating that plastic debris in aquatic environments primarily accumulates elements through surface adsorption from the surrounding environment, rather than through intrinsic properties [6,31,39]. The adsorbed environmentally derived elements, once released, can become bioavailable, potentially posing ecological and human health risks. The rate of this surface adsorption is influenced by both the physicochemical properties of the plastic itself and the environmental conditions and geochemical behavior of the elements in question [2,30].

PE was identified as the dominant polymer in this study, aligning with findings from a review by Dalvand and Hamidian [7] and echoing its widespread presence in wetland environments, typically followed by PP and PS. This abundance is often attributed to PE extensive use in the packaging industry and its role as a primary raw plastic material [40].

While this study did not find statistically significant differences (q < 0.05) in adsorbed element concentrations based on polymer type for all elements after FDR correction, notable trends emerged. PE exhibited the highest mean concentrations and the most outliers, suggesting a potentially greater overall affinity for element adsorption. Conversely, PS and PP, despite showing high adsorption levels in a previous study [6], displayed more consistent element adsorption behavior in this dataset, indicated by their smaller interquartile ranges. These findings highlight the complex interplay between polymeric structure, crystallinity, glass transition temperature (Tg), and adsorption. The classification of plastics as glassy (e.g., PES, PS, PET) or rubbery (e.g., PE, PP) based on Tg is crucial. Rubbery plastics, known for their larger free volumes, tend to exhibit higher metal adsorption capacities compared to dense, internally nanoporous glassy polymers [41], a trend that aligns with our observations and previous studies [30,42,43]. The pairwise post hoc test in “Element concentrations overview” Section, while limited in statistical power, indicated that the most prominent concentration differences tended to occur between polymers from these different groups (e.g., PES-PE and PS-PP), supporting this general trend.

Although pristine PE, PP, and PS are non-polar and hydrophobic, intrinsically resisting ion adsorption, the situation changes under environmental conditions. While PES and PET possess inherently polar and hydrophilic surfaces with active binding sites (COO and OH) for elements, our results, consistent with Abbasi et al. [6], suggest that the intrinsic surface charge of the pristine polymer may have a limited impact on element adsorption under ambient conditions, where factors like porosity and, crucially, environmental weathering likely play a larger role.

Given that the exact history of the plastic fragments collected in this study was unknown, the influence of environmental weathering becomes critical. Weathering processes, such as oxidation and chelation, create negative charges on polymer surfaces, enhancing adsorption capacity [32]. This is supported by Wang et al. [44], who demonstrated that aged polymers adsorbed more heavy metals than pristine polymers, likely due to increased surface area and oxygen-containing groups resulting from UV exposure. Moreover, the presence of organic matter, such as biofilms, attached to plastic pieces, exerts a strong control on element uptake [45,46].

The pH of the aqueous system further complicates adsorption dynamics. pH influences plastic surface charge [47], with acidic conditions promoting protonation and a positive charge, while neutral to alkaline conditions induce a negative charge, thus affecting electrostatic interactions with elements. This aligns with observations from Jian et al. [30] and Holmes et al. [48], who reported increased adsorption of Cu, Cd, and Pb onto plastic polymers with increasing pH. The seasonal pH fluctuations in the Curonian Lagoon, influenced by nutrient loads and biological activity [49,50], underscore the importance of considering such environmental factors.

**Fig 4:**
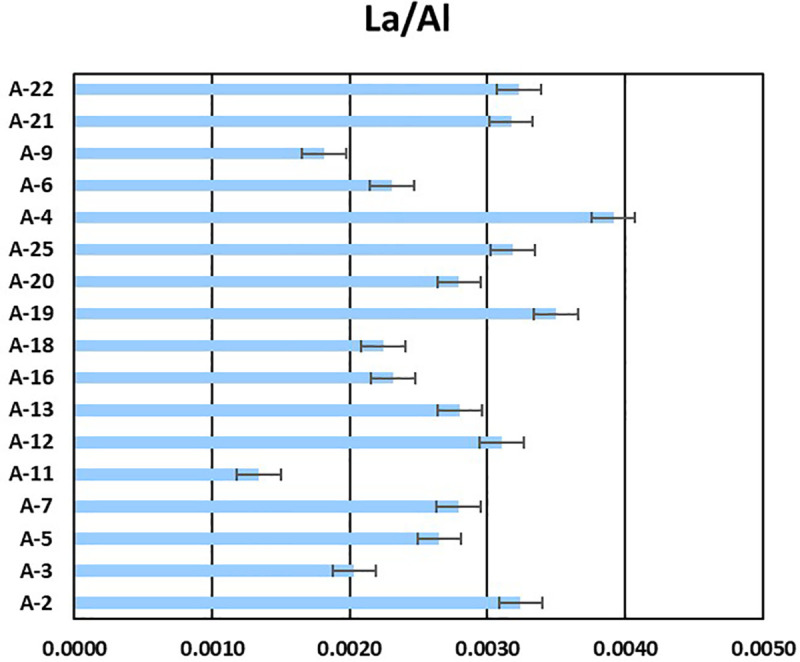
Al-to-La ratio in co-analyzed samples.

From a geochemical perspective, the charge-to-radius ratio of ions determines their mobility [51], influencing their adsorption potential and distribution. Elements with high ratios (e.g., Na, K, Ca, Mg, Sr, Ba) exhibit low mobility, while those with low ratios (e.g., Ce, Dy, Gd, La, Nd) have high [51]. Transition elements display intermediate mobility, sensitive to environmental conditions, as reflected in our correlation patterns (Fig 4). Ionic strength significantly impacts metal desorption from plastics by influencing competitive adsorption [29,52,53]. Jian et al. [30] observed that increasing potassium concentration initially increased and then decreased metal adsorption from a K+ solution. The strong positive correlations among intermediate elements (P, Si, Al, Cu, Mn, Ni, Fe, Zn, Fig 3) suggest that the presence of alkali ions may influence their co-adsorption. Elements like Ag, Sb, Cd, and S exhibit weak or negative correlations, indicating distinct reactivity, ion exchange capacity, and partitioning [54]. S, despite its abundance, showed no strong correlation, likely due to its high electronegativity favoring specific adsorption by polar polymers or biofilm components.

### Pollution sources and acknowledging uncertainties

The origin of elemental pollution in the Curonian Lagoon is of particular importance due to its specific location. The toxic elements Cd, Pb, and Ni showed moderate correlations with each other in this study but not with major geochemical groups. These elements have previously been associated with a high pollution load index in the Curonian Lagoon [27], which could be an indication of localized anthropogenic inputs near the Klaipėda Port leading to toxic element pollution and increased plastic adsorption (in line with the findings in “Comparison with previous studies” Section). However, the unknown and potentially varied sampling locations for our plastic debris limit our ability to definitively link these elements to a specific point source.

Among the trace metals, the strong correlation between Cu and Zn (ρ = 0.797) is a classic indication of non-exhaust emissions from traffic (such as brake and tire wear) [55]. Their significant correlations with crustal elements Al and Si further suggest their presence in polluted and suspended road dust [31]. In contrast, Sb showed a significant negative correlation with Mn (ρ = −0.619), highlighting a unique anthropogenic source (e.g., brake wear) that is isolated from the crustal manganese source [56,57]. Elements such as Ag showed weak negative correlations with REEs, indicating a distinct and potentially more isolated contamination pathway [58].

To further investigate geochemical provenance, we used the Al/La ratio, an established indicator for differentiating petroleum-related (Ratios of 0.012 and 0.004 for petroleum origin and 0.00056 and 0.000021 for natural origin in the study of Johnson et al. [59] and the study of Abbasi et al. [31], respectively). In our study (Fig 4), this ratio was greater than 0.003 for more than 40% of the samples, a value more closely related to an oil-related origin than a natural one. It is important to note, however, that these ratios were measured on plastics, which are a selective sink, and not on background environmental matrices (e.g., water, sediment). The lack of such background data introduces uncertainty, as we cannot directly compare the plastic-bound ratios to the ambient pool. Notwithstanding these uncertainties, the Al/La ratios leaning towards an oil signature are consistent with concerns about oil hydrocarbons (THCs) pollution in the Curonian Lagoon and the Baltic Sea, especially near the Klaipėda Strait due to maritime traffic and industrial activities [60,61].

REEs, classified as light or heavy, are emerging contaminants due to their industrial use [11,62]. They are found in consumer and environmental plastics, potentially through debris and dust contamination [11,63]. REE uptake can occur at functional sites like COOH, with light and heavy REEs following different complexation processes influenced by pH and competing ions [62]. The observed abundance of Ce, Y, La, and Nd is consistent with previous findings [11], suggesting widespread REE environmental presence. Ce and La, abundant in the upper continental crust [64], are common REEs reported in environmental studies [11,65] from sources like petroleum refineries and vehicle emissions [66–68]. Y is used in fluorescent powders and high-temperature alloys [69].

In spite of limitations including the absence of background samples like water and sediment (particularly for REEs), the lack of comparable data for bioavailable element adsorption in the initial modified BCR phase, the sample size, and the unidentified point sources, this research offers a valuable contribution. It represents a progressive step in employing REEs to trace the origin of pollutants adsorbed onto polymers, establishing a connection between elemental geochemistry and its relationship to plastic polymer adsorption. Furthermore, it paves the way for exploring the broader potential of these elements in environmental remediation efforts.

## Conclusion

In conclusion, this study highlights the intricate interplay of factors governing element absorption from the environment. The influence of co-existing ions, environmental conditions, and adsorbent surface characteristics underscores the complexity of this process. This necessitates further investigation into element adsorption on plastic debris within natural ecosystems, with particular emphasis on wetland environments given their susceptibility to contamination as enclosed systems. To facilitate robust comparisons of pollution levels across diverse ecosystems, future research should prioritize the adoption of standardized methodologies. Furthermore, the study emphasizes the potential of REEs analysis, when coupled with refined analytical techniques to minimize uncertainties, to more accurately trace pollution origins in environmental studies.

## Supporting information

S1 Table Kruskal-Wallis tests and Post-hoc pairwise comparisons using the Dwass-Steel-Critchlow-Fligner method, with FDR control. (DOCX)

S2 Table Spearman correlation analysis identified significant pairwise relationships among elements with >50% abundance adsorbed onto plastic polymers. (DOCX)

S1 Fig Graphical abstract. (JPG)
