# Correction: Geochemical signatures in plastic debris from the Curonian Lagoon, Lithuania

**DOI:** 10.1371/journal.pone.0347572

**Published:** 2026-04-17

**Authors:** Sajjad Abbasi, Neda Hashemi, Patryk Oleszczuk, Viktorija Sabaliauskaitė, Nerijus Dzingelevičius, Arūnas Balčiūnas, Rita Vaičekauskaitė, Reda Dzingelevičienė

In the Abstract section, there is an error in two to fourth sentences. The correct sentences are: Twenty-five plastic samples collected near the Curonian Lagoon in Lithuania were analyzed for 32 elements using an inductively coupled plasma-mass spectrometer (ICP-MS). Five common polymers (polyethylene, polypropylene, polystyrene, polyethersulfone, and polyethylene terephthalate) were identified, with polyethylene exhibiting the highest elemental uptake, followed by polypropylene and polystyrene. Correlation analysis suggested relationships between elemental uptake and geochemical behavior, with alkali and alkaline earth elements potentially enhancing the uptake of intermediate ions.

In the Abstract section, there is an error in the sixth sentence. The correct sentence is: Despite their low mobility, Rare Earth Elements (REEs) were used to infer sources of pollution, and the aluminum to lanthanum ratio was proposed as a potential indicator of possible anthropogenic pollution from industrial, petroleum, and vehicle emissions.

In the Element concentrations overview subsection of the Results, there is an error in the fifth sentence of the third paragraph. The correct sentence is: Cd was highest in PES (104.8 ± 6.59 µg kg^-1^), while Sb was highest in PET (40.1 µg kg^-1^ from one sample) (Fig 2).

In the Element concentrations overview subsection of the Results, there is an error in the seventh sentence of the third paragraph. The correct sentence is: PP exhibited the highest mean concentrations of Gd (23.19 ± 4.77 µg kg^-1^), Y (76.92 ± 18.53 µg kg^-1^) and Sm and Eu from one sample (32.26 and 4.30 µg kg^-1^) (Fig 3).

In the Correlation analysis subsection of the Results, there is an error in the third sentence of the first paragraph. The correct sentence is: Correlations that lost significance after FDR correction (p < 0.05, q < 0.1) are highlighted in yellow in Fig 4.

In the Pollution sources and acknowledging uncertainties subsection of the Discussion, there is an error in the second sentence of the third paragraph. The correct sentence is: In our study (Fig 5), this ratio was greater than 0.003 for more than 40% of the samples, a value more closely related to an oil-related origin than a natural one.

Figs 1–5 were uploaded incorrectly. Please see the correct Figs 1–5 and caption here.

There is an error in the caption for Table 1. The word ‘except’ is spelled incorrectly. Please see the correct Table 1 here.

In Table 2, the compounds “HNO_3_ + HCL” are missing from the ‘Methods’ column in row 11. Please see the correct Table 2 here.

In the Extraction and Digestion subsection of the Materials and methods, there is an error in the first sentence. The correct sentence is: To extract exchangeable metals from the polymer surface and associated biofilm, we used the first step of a modified BCR sequential extraction protocol [34] with 0.11 M acetic acid.

In the Extraction and Digestion subsection of the Materials and methods, there is an error in the sixth sentence. The correct sentence is: Following shaking, the samples were centrifuged at 4000 rpm for 20 min.

In the Instrumental Analysis subsection of the Materials and methods, there is an error in the second sentence. The correct sentence is: Elemental analysis, including Group 1 and 2, transition elements, and REEs, was performed using inductively coupled plasma-mass spectrometer (ICP-MS).

**Fig 1:**
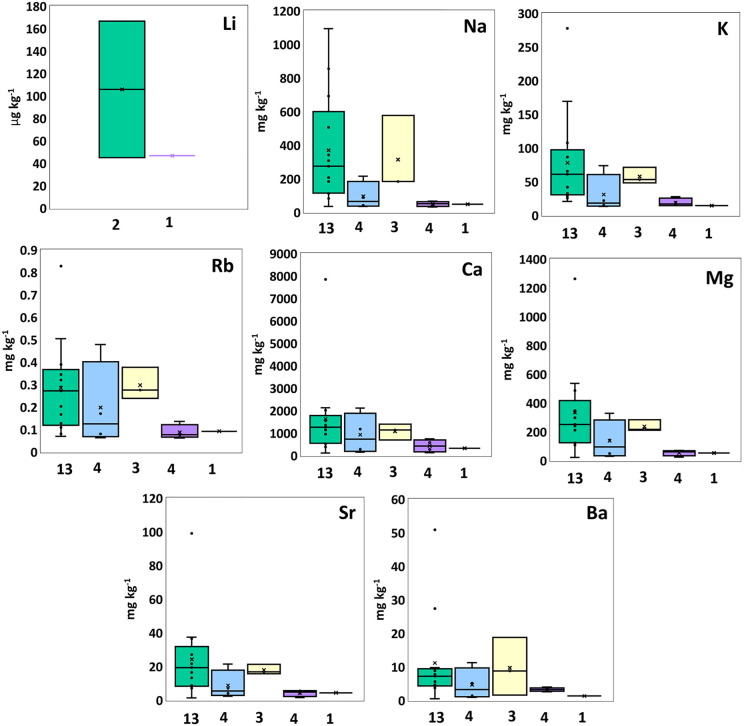
Box plots summarize group one and two element concentrations in PE (green), PP (blue), PS (yellow), PES (purple), and PET (black), displaying median, mean (×), interquartile range, minimum, maximum, and outliers.

**Fig 2:**
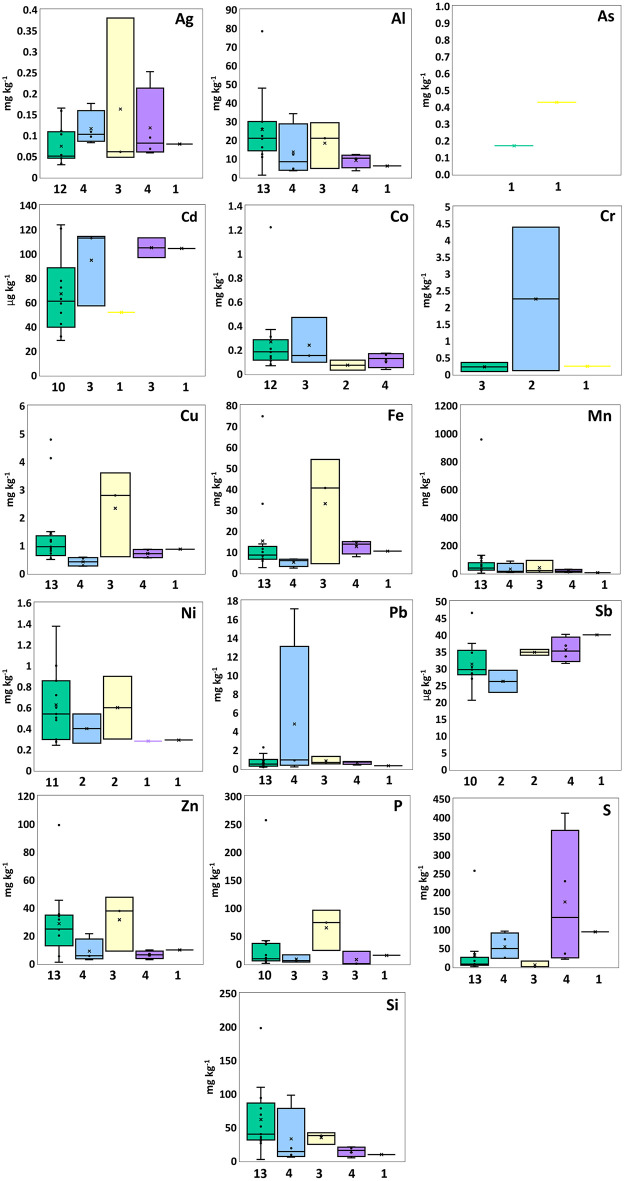
Box plots summarize intermediate elements concentrations in PE (green), PP (blue), PS (yellow), PES (purple), and PET (black), displaying median, mean (×), interquartile range, minimum, maximum, and outliers.

**Fig 3:**
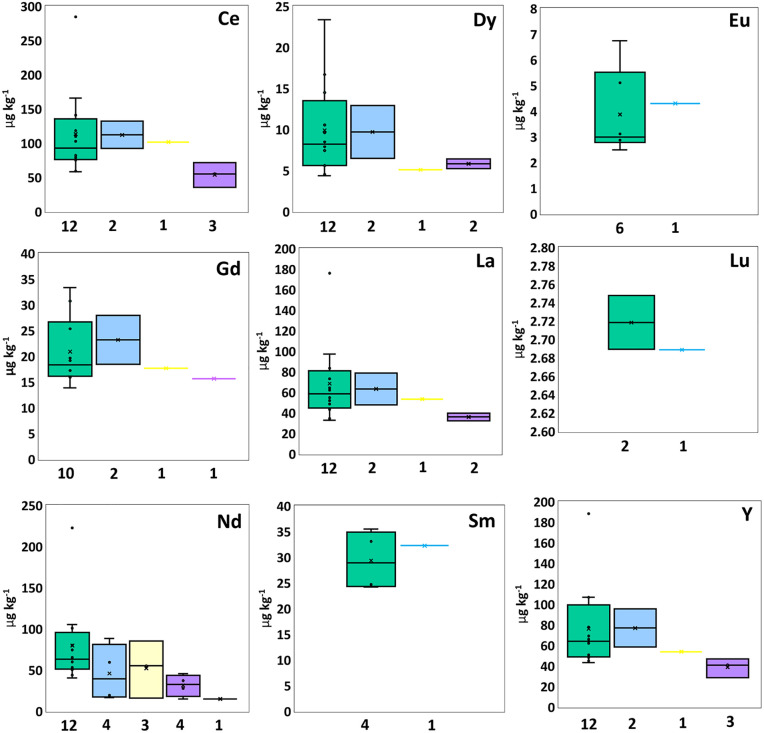
Box plots summarize rare earth element (REEs) concentrations in PE (green), PP (blue), PS (yellow), PES (purple), and PET (black), displaying median, mean (×), interquartile range, minimum, maximum, and outliers.

**Fig 4:**
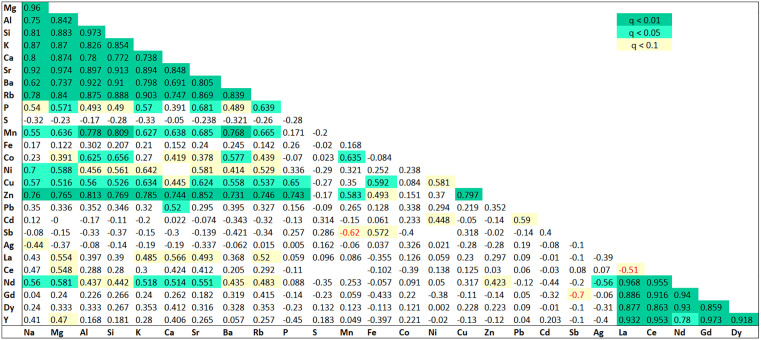
The correlation matrix of element concentrations highlights significant pairwise relationships (abundance > 50%). Significance levels: q < 0.01, dark green; q < 0.05, light green; q < 0.1, yellow; negative correlations, red.

**Fig 5:**
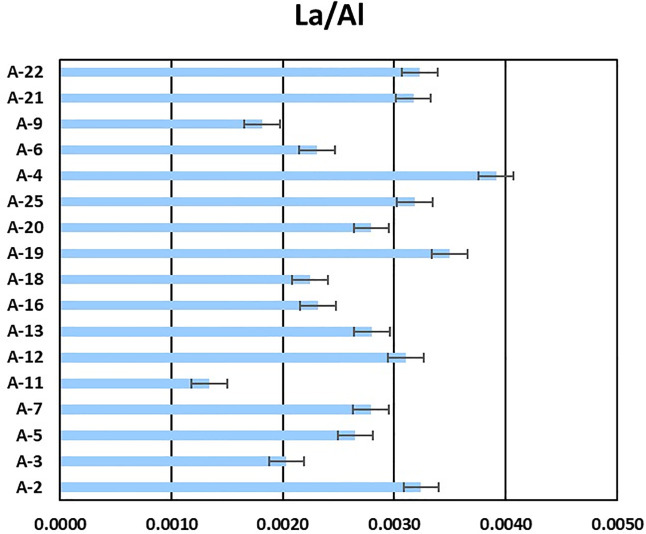
Al-to-La ratio in co-analyzed samples.

**Table 1:** Detection limits (MDL; µg L^-1^) of elements, normalized to plastic weight and solution volume (mg kg^-1^) except for Li, Cd, Sb, and REEs (µg kg^-1^).

Elements	MDL (µg L^-1^)	MDL (µg kg^-1^)	Elements	MDL (µg L^-1^)	MDL (µg kg^-1^)
Ag	1	0.03	Li	1	27.65
Al	100	0.29	Cd	1	27.15
As	1	0.03	Sb	1	25.36
Ba	1	0.03	Ce	1	28.15
Ca	100	0.29	Dy	0.1	2.82
Co	1	0.03	Eu	0.1	2.51
Cr	1	0.03	Gd	0.5	13.29
Cu	100	0.29	La	1	28.16
Fe	100	0.29	Lu	0.1	2.63
K	100	0.29	Nd	0.5	14.68
Mg	100	0.29	Sm	1	25.34
Mn	100	0.29	Y	1	28.15
Na	100	0.29			
Ni	100	0.28			
P	100	0.29			
Pb	1	0.03			
Rb	1	0.03			
S	100	0.29			
Si	100	0.29			
Sr	100	0.29			
Zn	100	0.29			

**Table 2:** Comparison of the concentrations of the studied elements with other previous studies.

Concentration (mg kg^-1^)												Method	Environment	Studies
As	Co	Cr	Cu	Fe	Mn	Ni	Pb	Zn	Ca	Mg	Ba			
0.17	0.27	0.22	1.44	15.27	115.77	0.62	0.73	28.70	1630.50	333.60	11.15	acetic acid	Curonian Lagoon, Lithuania	This Study
ND	0.24	2.25	0.42	5.18	30.08	0.40	4.80	8.97	929.47	137.12	4.66			
0.43	0.07	0.24	2.33	33.07	39.53	0.60	0.84	31.44	1074.73	236.60	9.73			
ND	0.12	ND	0.71	12.57	15.95	0.28	0.66	6.38	425.43	53.24	3.23			
ND	0.03	ND	0.87	10.42	3.47	0.29	0.32	9.84	319.65	52.12	1.34			
0.30	0.14	0.90	1.15	15.30	40.96	0.44	1.47	17.07	875.96	162.53	6.02			
	0.03	0.02	0.07	2.78	7.58	0.24	0.03	3.63				acetic acid	Various environments of Lublin Province, Poland	Abbasi et al. [6]
	0.17	0.02	0.45	5.39	39.46	0.43	0.31	18.30						
	0.08	0.03	0.35	3.75	31.85	0.06	0.09							
	0.09	0.03	0.29	3.97	26.30	0.25	0.15	10.97						
0.13		0.18	1.11		1.04	1.15	0.59	8.70			19.11	HNO_3_ + HCL	Australian coastline	Carbery et al. [38]
			3.20	202.25			0.86	21.16				HNO_3_ + 30% HCL	The coast of Bahia, Brazil	Souza et al. [29]
	0.02	0.37	0.73	63.20	6.17	0.09	1.04	11.82				HNO_3_ + 10% HCL	The beaches of south-west England	Holmes et al. [15]
	0.14	0.43	0.42	34.40	0.71	0.30	0.11	0.20				0.03 M HCl	laboratory conditions	Turner &Holmes [37]
									50000	5000		70% HNO_3_	Australian coastline	Lee et al. [30]
**Concentration (µg kg**^**-1**^)												**Method**	**Environment**	**Studies**
**Cd**	**Ce**	**Dy**	**Eu**	**Gd**	**La**	**Lu**	**Nd**	**Sm**	**Y**					
67.10	113.14	9.89	3.87	20.89	68.46	2.72	79.73	29.36	76.23			acetic acid	Curonian Lagoon, Lithuania	This Study
94.74	112.07	9.70	4.30	23.19	63.26	2.69	45.88	32.26	76.92					
ND	101.50	5.09	ND	17.66	53.29	ND	52.07	ND	53.89					
104.80	53.95	5.82	ND	15.64	35.92	ND	31.28	ND	38.79					
ND	ND	ND	ND	ND	ND	ND	14.77	ND	ND					
88.88	95.16	7.62	4.09	19.35	55.24	2.70	44.74	30.81	61.46					
9.01												acetic acid	Various environments of Lublin Province, Poland	Abbasi et al. [6]
822.80														
ND														
415.90														
210.53												HNO_3_ + HCL	Australian coastline	Carbery et al. [38]
440	371.00	16.86	6.34	24.40	200.50	1.21	159.35	24.64	101.70			HNO_3_ + 30% HCL	The coast of Bahia, Brazil	Souza et al. [29]
38.46													The beaches of south-west England	Holmes et al. [15]
5.00	540	120	99	120	880	8	380	160	340			HNO_3_ + 30% HCL	laboratory conditions	Turner et al. [11]
