# Supplementary material for: Geochemical signatures in plastic debris from the Curonian Lagoon, Lithuania
Source: PLoS One. 2026 Feb 2;21(2):e0340582. doi: 10.1371/journal.pone.0340582 (PMC12863676; doi:10.1371/journal.pone.0340582)
Supplement: S1 Table — (DOCX) [file pone.0340582.s001.docx]

S1 Table. Kruskal-Wallis tests and Post-hoc pairwise comparisons using the Dwass-Steel-Critchlow-Fligner method, with FDR control.

| **a. Independent-Samples Kruskal-Wallis Test** | | | | | | | |
| --- | --- | --- | --- | --- | --- | --- | --- |
| Elements | p-value |  | Elements | p-value |  | Elements | p-value |
| Ag | 0.152 |  | Eu | 0.54 |  | Ni | 0.286 |
| Al | 0.122 |  | Fe | 0.07 |  | P | 0.028 |
| As | 0.344 |  | Gd | 0.136 |  | Pb | 0.676 |
| Ba | 0.204 |  | K | 0.025 |  | Rb | 0.076 |
| Ca | 0.255 |  | La | 0.027 |  | S | 0.011 |
| Cd | 0.353 |  | Li | 0.661 |  | Sb | 0.065 |
| Ce | 0.52 |  | Lu | 0.68 |  | Si | 0.053 |
| Co | 0.235 |  | Mg | 0.052 |  | Sm | 0.87 |
| Cr | 0.407 |  | Mn | 0.215 |  | Sr | 0.038 |
| Cu | 0.03 |  | Na | 0.024 |  | Y | 0.034 |
| Dy | 0.033 |  | Nd | 0.072 |  | Zn | 0.08 |
| **b. Pairwise Comparisons of Polymer** | | | | | | | |
| Cu | p-value | rank | q-value (FDR) | Sr | p-value | rank | q-value (FDR) |
| PP-PS | 0.011 | 2 | 0.033 | PES-PE | 0.01 | 1 | 0.06 |
| PP-PE | 0.009 | 1 | 0.054 | PES-PS | 0.056 | 2 | 0.168 |
| PP-PES | 0.211 | 4 | 0.3165 | PP-PE | 0.089 | 3 | 0.178 |
| PES-PE | 0.28 | 5 | 0.336 | PP-PS | 0.206 | 4 | 0.309 |
| PES-PS | 0.17 | 3 | 0.34 | PES-PP | 0.484 | 5 | 0.5808 |
| PE-PS | 0.5 | 6 | 0.5 | PS-PE | 0.991 | 6 | 0.991 |
| K |  |  |  | Dy |  |  |  |
| PES-PE | 0.01 | 1 | 0.06 | PS-PE | 0.016 | 1 | 0.096 |
| PP-PE | 0.056 | 3 | 0.112 | PES-PE | 0.034 | 2 | 0.102 |
| PES-PS | 0.042 | 2 | 0.126 | PP-PE | 0.157 | 3 | 0.314 |
| PP-PS | 0.127 | 4 | 0.1905 | PS-PP | 0.338 | 4 | 0.507 |
| PES-PP | 0.582 | 5 | 0.6984 | PS-PES | 0.673 | 6 | 0.673 |
| PE-PS | 0.905 | 6 | 0.905 | PES-PP | 0.563 | 5 | 0.6756 |
| Na |  |  |  | La |  |  |  |
| PES-PE | 0.007 | 1 | 0.042 | PES-PE | 0.011 | 1 | 0.066 |
| PP-PE | 0.06 | 3 | 0.12 | PS-PE | 0.048 | 2 | 0.144 |
| PES-PS | 0.05 | 2 | 0.15 | PP-PE | 0.112 | 3 | 0.224 |
| PP-PS | 0.175 | 4 | 0.2625 | PES-PP | 0.43 | 4 | 0.645 |
| PES-PP | 0.516 | 5 | 0.6192 | PS-PP | 0.638 | 5 | 0.7656 |
| PS-PE | 0.95 | 6 | 0.95 | PES-PS | 0.795 | 6 | 0.795 |
| P |  |  |  | Y |  |  |  |
| PS-PP | 0.009 | 1 | 0.027 | PES-PE | 0.01 | 1 | 0.06 |
| PE-PP | 0.055 | 2 | 0.0825 | PS-PE | 0.064 | 2 | 0.192 |
| PS-PE | 0.159 | 3 | 0.159 | PP-PE | 0.158 | 3 | 0.316 |
| S |  |  |  | PES-PP | 0.346 | 4 | 0.519 |
| PS-PES | 0.006 | 1 | 0.036 | PES-PS | 0.71 | 6 | 0.71 |
| PS-PP | 0.014 | 2 | 0.042 | PS-PP | 0.616 | 5 | 0.7392 |
| PE-PES | 0.025 | 3 | 0.05 |  |  |  |  |
| PE-PP | 0.062 | 4 | 0.093 |  |  |  |  |
| PS-PE | 0.201 | 5 | 0.2412 |  |  |  |  |
| PP-PES | 0.764 | 6 | 0.764 |  |  |  |  |
| Asymptotic significances (2-sided tests) are displayed. The significance level is .050. | | | | | | |  |
