# Supplementary material for: Geochemical signatures in plastic debris from the Curonian Lagoon, Lithuania
Source: PLoS One. 2026 Feb 2;21(2):e0340582. doi: 10.1371/journal.pone.0340582 (PMC12863676; doi:10.1371/journal.pone.0340582)
Supplement: S2 Table — (DOCX) [file pone.0340582.s002.docx]

S2 Table. Spearman correlation analysis identified significant pairwise relationships among elements with >50% abundance adsorbed onto plastic polymers.

| **Confidence Intervals of Spearman's rho** | | | | | | | |  |
| --- | --- | --- | --- | --- | --- | --- | --- | --- |
| Elements | Spearman's rho | Significance(2-tailed) | rank | q-value (FDR) | 95% Confidence Intervals (2-tailed) ^a,b^ | | Number of samples |  |
|  |  |  |  |  |  |  |  |  |
|  |  |  |  |  | Lower | Upper |  |  |
| Na - K | 0.865 | 0.000 | 21 | 0.000 | 0.707 | 0.940 | 25 |  |
| Na - Rb | 0.778 | 0.000 | 45 | 0.000 | 0.544 | 0.900 | 25 |  |
| Na - Ca | 0.801 | 0.000 | 38 | 0.000 | 0.585 | 0.911 | 25 |  |
| Na - Mg | 0.958 | 0.000 | 3 | 0.000 | 0.905 | 0.982 | 25 |  |
| Na - Sr | 0.924 | 0.000 | 4 | 0.000 | 0.829 | 0.967 | 25 |  |
| Na - Ba | 0.622 | 0.001 | 72 | 0.004 | 0.290 | 0.821 | 25 |  |
| Na - P | 0.538 | 0.014 | 105 | 0.048 | 0.112 | 0.797 | 20 |  |
| Na - S | -0.321 | 0.118 | 165 | 0.251 | -0.643 | 0.097 | 25 |  |
| Na - Si | 0.806 | 0.000 | 36 | 0.000 | 0.595 | 0.913 | 25 |  |
| Na - Ag | -0.438 | 0.032 | 120 | 0.094 | -0.721 | -0.030 | 24 |  |
| Na - Al | 0.748 | 0.000 | 53 | 0.000 | 0.491 | 0.885 | 25 |  |
| Na - Cd | 0.120 | 0.646 | 291 | 0.779 | -0.396 | 0.578 | 17 |  |
| Na - Co | 0.230 | 0.316 | 229 | 0.485 | -0.237 | 0.610 | 21 |  |
| Na - Cu | 0.568 | 0.003 | 83 | 0.013 | 0.211 | 0.791 | 25 |  |
| Na - Fe | 0.168 | 0.423 | 253 | 0.587 | -0.255 | 0.537 | 25 |  |
| Na - Mn | 0.545 | 0.005 | 88 | 0.019 | 0.179 | 0.778 | 25 |  |
| Na - Ni | 0.701 | 0.002 | 75 | 0.008 | 0.318 | 0.887 | 17 |  |
| Na - Pb | 0.345 | 0.092 | 148 | 0.217 | -0.071 | 0.658 | 25 |  |
| Na - Sb | -0.075 | 0.759 | 312 | 0.854 | -0.523 | 0.404 | 19 |  |
| Na - Zn | 0.757 | 0.000 | 52 | 0.000 | 0.507 | 0.889 | 25 |  |
| Na - Ce | 0.465 | 0.052 | 131 | 0.138 | -0.017 | 0.772 | 18 |  |
| Na - Dy | 0.243 | 0.348 | 234 | 0.522 | -0.284 | 0.657 | 17 |  |
| Na - Gd | 0.037 | 0.899 | 336 | 0.939 | -0.516 | 0.569 | 14 |  |
| Na - La | 0.431 | 0.084 | 139 | 0.212 | -0.078 | 0.762 | 17 |  |
| Na - Nd | 0.558 | 0.005 | 86 | 0.019 | 0.188 | 0.790 | 24 |  |
| Na - Y | 0.408 | 0.093 | 149 | 0.219 | -0.088 | 0.741 | 18 |  |
| K - Rb | 0.903 | 0.000 | 10 | 0.000 | 0.785 | 0.958 | 25 |  |
| K - Ca | 0.738 | 0.000 | 58 | 0.000 | 0.474 | 0.880 | 25 |  |
| K - Mg | 0.870 | 0.000 | 19 | 0.000 | 0.718 | 0.943 | 25 |  |
| K - Sr | 0.894 | 0.000 | 13 | 0.000 | 0.766 | 0.954 | 25 |  |
| K - Ba | 0.798 | 0.000 | 39 | 0.000 | 0.581 | 0.909 | 25 |  |
| K - P | 0.570 | 0.009 | 98 | 0.031 | 0.157 | 0.813 | 20 |  |
| K - S | -0.326 | 0.112 | 159 | 0.246 | -0.646 | 0.091 | 25 |  |
| K - Si | 0.854 | 0.000 | 23 | 0.000 | 0.686 | 0.935 | 25 |  |
| K - Ag | -0.187 | 0.382 | 244 | 0.549 | -0.558 | 0.246 | 24 |  |
| K - Al | 0.826 | 0.000 | 31 | 0.000 | 0.633 | 0.923 | 25 |  |
| K - Cd | -0.196 | 0.451 | 259 | 0.611 | -0.628 | 0.328 | 17 |  |
| K - Co | 0.270 | 0.236 | 207 | 0.401 | -0.196 | 0.637 | 21 |  |
| K - Cu | 0.634 | 0.001 | 69 | 0.003 | 0.307 | 0.827 | 25 |  |
| K - Fe | 0.210 | 0.314 | 228 | 0.483 | -0.214 | 0.567 | 25 |  |
| K - Mn | 0.627 | 0.001 | 70 | 0.004 | 0.297 | 0.823 | 25 |  |
| K - Ni | 0.642 | 0.005 | 90 | 0.021 | 0.219 | 0.862 | 17 |  |
| K - Pb | 0.320 | 0.119 | 167 | 0.250 | -0.098 | 0.642 | 25 |  |
| K - Sb | -0.151 | 0.538 | 273 | 0.691 | -0.576 | 0.339 | 19 |  |
| K - Zn | 0.785 | 0.000 | 41 | 0.000 | 0.557 | 0.903 | 25 |  |
| K - Ce | 0.300 | 0.226 | 203 | 0.391 | -0.208 | 0.681 | 18 |  |
| K - Dy | 0.353 | 0.165 | 183 | 0.316 | -0.169 | 0.720 | 17 |  |
| K - Gd | 0.240 | 0.409 | 247 | 0.582 | -0.349 | 0.692 | 14 |  |
| K - La | 0.485 | 0.048 | 128 | 0.132 | -0.009 | 0.789 | 17 |  |
| K - Nd | 0.518 | 0.009 | 99 | 0.034 | 0.133 | 0.768 | 24 |  |
| K - Y | 0.280 | 0.261 | 218 | 0.420 | -0.230 | 0.669 | 18 |  |
| Rb - Ca | 0.747 | 0.000 | 54 | 0.000 | 0.490 | 0.885 | 25 |  |
| Rb - Mg | 0.840 | 0.000 | 29 | 0.000 | 0.659 | 0.929 | 25 |  |
| Rb - Sr | 0.869 | 0.000 | 20 | 0.000 | 0.716 | 0.943 | 25 |  |
| Rb - Ba | 0.839 | 0.000 | 30 | 0.000 | 0.657 | 0.929 | 25 |  |
| Rb - P | 0.639 | 0.002 | 80 | 0.011 | 0.261 | 0.847 | 20 |  |
| Rb - S | -0.260 | 0.209 | 199 | 0.369 | -0.602 | 0.163 | 25 |  |
| Rb - Si | 0.888 | 0.000 | 14 | 0.000 | 0.753 | 0.951 | 25 |  |
| Rb - Ag | 0.015 | 0.945 | 345 | 0.962 | -0.402 | 0.426 | 24 |  |
| Rb - Al | 0.875 | 0.000 | 17 | 0.000 | 0.728 | 0.945 | 25 |  |
| Rb - Cd | -0.319 | 0.213 | 200 | 0.373 | -0.701 | 0.206 | 17 |  |
| Rb - Co | 0.439 | 0.047 | 127 | 0.129 | -0.005 | 0.738 | 21 |  |
| Rb - Cu | 0.537 | 0.006 | 91 | 0.022 | 0.168 | 0.774 | 25 |  |
| Rb - Fe | 0.142 | 0.500 | 265 | 0.662 | -0.280 | 0.517 | 25 |  |
| Rb - Mn | 0.665 | 0.000 | 66 | 0.002 | 0.356 | 0.843 | 25 |  |
| Rb - Ni | 0.529 | 0.029 | 117 | 0.087 | 0.050 | 0.811 | 17 |  |
| Rb - Pb | 0.327 | 0.111 | 158 | 0.246 | -0.091 | 0.647 | 25 |  |
| Rb - Sb | -0.337 | 0.158 | 180 | 0.309 | -0.694 | 0.153 | 19 |  |
| Rb - Zn | 0.746 | 0.000 | 55 | 0.000 | 0.488 | 0.884 | 25 |  |
| Rb - Ce | 0.292 | 0.240 | 209 | 0.402 | -0.217 | 0.676 | 18 |  |
| Rb - Dy | 0.353 | 0.165 | 183 | 0.316 | -0.169 | 0.720 | 17 |  |
| Rb - Gd | 0.415 | 0.140 | 174 | 0.282 | -0.165 | 0.782 | 14 |  |
| Rb - La | 0.520 | 0.033 | 121 | 0.094 | 0.036 | 0.806 | 17 |  |
| Rb - Nd | 0.483 | 0.017 | 108 | 0.054 | 0.087 | 0.748 | 24 |  |
| Rb - Y | 0.257 | 0.303 | 227 | 0.469 | -0.253 | 0.655 | 18 |  |
| Ca - Mg | 0.874 | 0.000 | 18 | 0.000 | 0.725 | 0.945 | 25 |  |
| Ca - Sr | 0.848 | 0.000 | 25 | 0.000 | 0.674 | 0.933 | 25 |  |
| Ca - Ba | 0.691 | 0.000 | 62 | 0.001 | 0.396 | 0.856 | 25 |  |
| Ca - P | 0.391 | 0.088 | 143 | 0.217 | -0.076 | 0.717 | 20 |  |
| Ca - S | -0.054 | 0.798 | 320 | 0.876 | -0.450 | 0.360 | 25 |  |
| Ca - Si | 0.772 | 0.000 | 47 | 0.000 | 0.534 | 0.897 | 25 |  |
| Ca - Ag | -0.193 | 0.366 | 237 | 0.542 | -0.562 | 0.240 | 24 |  |
| Ca - Al | 0.780 | 0.000 | 44 | 0.000 | 0.548 | 0.901 | 25 |  |
| Ca - Cd | 0.022 | 0.933 | 341 | 0.960 | -0.476 | 0.509 | 17 |  |
| Ca - Co | 0.419 | 0.058 | 132 | 0.155 | -0.029 | 0.727 | 21 |  |
| Ca - Cu | 0.445 | 0.026 | 113 | 0.081 | 0.048 | 0.720 | 25 |  |
| Ca - Fe | 0.152 | 0.467 | 260 | 0.631 | -0.270 | 0.525 | 25 |  |
| Ca - Mn | 0.638 | 0.001 | 67 | 0.003 | 0.313 | 0.829 | 25 |  |
| Ca - Ni | 0.321 | 0.209 | 196 | 0.374 | -0.204 | 0.702 | 17 |  |
| Ca - Pb | 0.520 | 0.008 | 95 | 0.028 | 0.145 | 0.764 | 25 |  |
| Ca - Sb | -0.302 | 0.209 | 198 | 0.371 | -0.673 | 0.191 | 19 |  |
| Ca - Zn | 0.744 | 0.000 | 56 | 0.000 | 0.484 | 0.883 | 25 |  |
| Ca - Ce | 0.424 | 0.079 | 137 | 0.203 | -0.068 | 0.750 | 18 |  |
| Ca - Dy | 0.412 | 0.101 | 154 | 0.229 | -0.101 | 0.752 | 17 |  |
| Ca - Gd | 0.262 | 0.366 | 238 | 0.540 | -0.328 | 0.705 | 14 |  |
| Ca - La | 0.566 | 0.018 | 109 | 0.057 | 0.102 | 0.828 | 17 |  |
| Ca - Nd | 0.514 | 0.010 | 100 | 0.036 | 0.127 | 0.765 | 24 |  |
| Ca - Y | 0.406 | 0.095 | 150 | 0.222 | -0.090 | 0.740 | 18 |  |
| Mg - Sr | 0.974 | 0.000 | 1 | 0.000 | 0.939 | 0.989 | 25 |  |
| Mg - Ba | 0.737 | 0.000 | 59 | 0.000 | 0.473 | 0.880 | 25 |  |
| Mg - P | 0.571 | 0.008 | 97 | 0.031 | 0.159 | 0.814 | 20 |  |
| Mg - S | -0.225 | 0.280 | 223 | 0.441 | -0.578 | 0.199 | 25 |  |
| Mg - Si | 0.883 | 0.000 | 16 | 0.000 | 0.744 | 0.949 | 25 |  |
| Mg - Ag | -0.365 | 0.079 | 136 | 0.205 | -0.677 | 0.057 | 24 |  |
| Mg - Al | 0.842 | 0.000 | 27 | 0.000 | 0.662 | 0.930 | 25 |  |
| Mg - Cd | -0.002 | 0.993 | 350 | 0.995 | -0.494 | 0.491 | 17 |  |
| Mg - Co | 0.391 | 0.080 | 138 | 0.203 | -0.063 | 0.711 | 21 |  |
| Mg - Cu | 0.516 | 0.008 | 96 | 0.030 | 0.140 | 0.762 | 25 |  |
| Mg - Fe | 0.122 | 0.560 | 276 | 0.713 | -0.298 | 0.503 | 25 |  |
| Mg - Mn | 0.636 | 0.001 | 68 | 0.003 | 0.311 | 0.828 | 25 |  |
| Mg - Ni | 0.588 | 0.013 | 103 | 0.044 | 0.135 | 0.838 | 17 |  |
| Mg - Pb | 0.336 | 0.100 | 153 | 0.230 | -0.080 | 0.653 | 25 |  |
| Mg - Sb | -0.147 | 0.547 | 275 | 0.698 | -0.574 | 0.342 | 19 |  |
| Mg - Zn | 0.765 | 0.000 | 51 | 0.000 | 0.520 | 0.893 | 25 |  |
| Mg - Ce | 0.548 | 0.019 | 110 | 0.059 | 0.094 | 0.813 | 18 |  |
| Mg - Dy | 0.333 | 0.191 | 192 | 0.349 | -0.190 | 0.709 | 17 |  |
| Mg - Gd | 0.240 | 0.409 | 247 | 0.582 | -0.349 | 0.692 | 14 |  |
| Mg - La | 0.554 | 0.021 | 112 | 0.066 | 0.085 | 0.822 | 17 |  |
| Mg - Nd | 0.581 | 0.003 | 82 | 0.012 | 0.220 | 0.802 | 24 |  |
| Mg - Y | 0.470 | 0.049 | 129 | 0.134 | -0.012 | 0.774 | 18 |  |
| Sr - Ba | 0.805 | 0.000 | 37 | 0.000 | 0.594 | 0.913 | 25 |  |
| Sr - P | 0.681 | 0.001 | 73 | 0.005 | 0.329 | 0.867 | 20 |  |
| Sr - S | -0.238 | 0.253 | 217 | 0.409 | -0.587 | 0.186 | 25 |  |
| Sr - Si | 0.913 | 0.000 | 7 | 0.000 | 0.806 | 0.962 | 25 |  |
| Sr - Ag | -0.337 | 0.107 | 157 | 0.239 | -0.659 | 0.089 | 24 |  |
| Sr - Al | 0.897 | 0.000 | 12 | 0.000 | 0.772 | 0.955 | 25 |  |
| Sr - Cd | -0.074 | 0.779 | 316 | 0.865 | -0.546 | 0.435 | 17 |  |
| Sr - Co | 0.378 | 0.091 | 147 | 0.218 | -0.078 | 0.703 | 21 |  |
| Sr - Cu | 0.624 | 0.001 | 71 | 0.004 | 0.292 | 0.822 | 25 |  |
| Sr - Fe | 0.240 | 0.248 | 214 | 0.407 | -0.183 | 0.588 | 25 |  |
| Sr - Mn | 0.685 | 0.000 | 63 | 0.001 | 0.388 | 0.854 | 25 |  |
| Sr - Ni | 0.581 | 0.014 | 106 | 0.048 | 0.124 | 0.835 | 17 |  |
| Sr - Pb | 0.295 | 0.153 | 179 | 0.300 | -0.126 | 0.625 | 25 |  |
| Sr - Sb | -0.139 | 0.571 | 277 | 0.724 | -0.568 | 0.350 | 19 |  |
| Sr - Zn | 0.852 | 0.000 | 24 | 0.000 | 0.683 | 0.935 | 25 |  |
| Sr - Ce | 0.412 | 0.090 | 145 | 0.217 | -0.083 | 0.744 | 18 |  |
| Sr - Dy | 0.316 | 0.216 | 201 | 0.378 | -0.209 | 0.700 | 17 |  |
| Sr - Gd | 0.182 | 0.533 | 272 | 0.687 | -0.400 | 0.660 | 14 |  |
| Sr - La | 0.493 | 0.045 | 126 | 0.124 | 0.000 | 0.793 | 17 |  |
| Sr - Nd | 0.551 | 0.005 | 89 | 0.021 | 0.178 | 0.786 | 24 |  |
| Sr - Y | 0.265 | 0.287 | 224 | 0.450 | -0.244 | 0.660 | 18 |  |
| Ba - P | 0.489 | 0.029 | 116 | 0.087 | 0.045 | 0.771 | 20 |  |
| Ba - S | -0.321 | 0.118 | 165 | 0.251 | -0.643 | 0.097 | 25 |  |
| Ba - Si | 0.910 | 0.000 | 9 | 0.000 | 0.800 | 0.961 | 25 |  |
| Ba - Ag | -0.062 | 0.774 | 315 | 0.863 | -0.464 | 0.361 | 24 |  |
| Ba - Al | 0.922 | 0.000 | 5 | 0.000 | 0.826 | 0.966 | 25 |  |
| Ba - Cd | -0.343 | 0.178 | 187 | 0.333 | -0.715 | 0.180 | 17 |  |
| Ba - Co | 0.577 | 0.006 | 93 | 0.023 | 0.180 | 0.812 | 21 |  |
| Ba - Cu | 0.558 | 0.004 | 85 | 0.016 | 0.197 | 0.786 | 25 |  |
| Ba - Fe | 0.245 | 0.237 | 208 | 0.400 | -0.178 | 0.592 | 25 |  |
| Ba - Mn | 0.768 | 0.000 | 49 | 0.000 | 0.526 | 0.895 | 25 |  |
| Ba - Ni | 0.414 | 0.098 | 152 | 0.227 | -0.098 | 0.753 | 17 |  |
| Ba - Pb | 0.395 | 0.051 | 130 | 0.137 | -0.013 | 0.690 | 25 |  |
| Ba - Sb | -0.421 | 0.073 | 134 | 0.190 | -0.741 | 0.055 | 19 |  |
| Ba - Zn | 0.731 | 0.000 | 60 | 0.000 | 0.462 | 0.877 | 25 |  |
| Ba - Ce | 0.205 | 0.414 | 251 | 0.578 | -0.303 | 0.623 | 18 |  |
| Ba - Dy | 0.328 | 0.198 | 194 | 0.358 | -0.196 | 0.707 | 17 |  |
| Ba - Gd | 0.319 | 0.267 | 221 | 0.424 | -0.271 | 0.735 | 14 |  |
| Ba - La | 0.368 | 0.147 | 178 | 0.289 | -0.152 | 0.728 | 17 |  |
| Ba - Nd | 0.435 | 0.034 | 123 | 0.096 | 0.025 | 0.719 | 24 |  |
| Ba - Y | 0.057 | 0.823 | 325 | 0.889 | -0.434 | 0.521 | 18 |  |
| P - S | -0.281 | 0.230 | 204 | 0.395 | -0.652 | 0.198 | 20 |  |
| P - Si | 0.490 | 0.028 | 115 | 0.086 | 0.047 | 0.772 | 20 |  |
| P - Ag | 0.005 | 0.983 | 349 | 0.989 | -0.461 | 0.470 | 19 |  |
| P - Al | 0.493 | 0.027 | 114 | 0.083 | 0.051 | 0.774 | 20 |  |
| P - Cd | -0.132 | 0.668 | 295 | 0.794 | -0.647 | 0.466 | 13 |  |
| P - Co | -0.071 | 0.786 | 317 | 0.871 | -0.544 | 0.437 | 17 |  |
| P - Cu | 0.650 | 0.002 | 77 | 0.009 | 0.278 | 0.852 | 20 |  |
| P - Fe | 0.260 | 0.268 | 222 | 0.424 | -0.220 | 0.639 | 20 |  |
| P - Mn | 0.171 | 0.470 | 262 | 0.630 | -0.306 | 0.580 | 20 |  |
| P - Ni | 0.336 | 0.240 | 210 | 0.401 | -0.253 | 0.744 | 14 |  |
| P - Pb | 0.156 | 0.510 | 269 | 0.666 | -0.320 | 0.570 | 20 |  |
| P - Sb | 0.257 | 0.319 | 230 | 0.486 | -0.269 | 0.665 | 17 |  |
| P - Zn | 0.743 | 0.000 | 65 | 0.001 | 0.436 | 0.895 | 20 |  |
| P - Ce | -0.111 | 0.694 | 300 | 0.813 | -0.600 | 0.439 | 15 |  |
| P - Dy | -0.231 | 0.427 | 255 | 0.588 | -0.688 | 0.357 | 14 |  |
| P - Gd | -0.136 | 0.689 | 299 | 0.809 | -0.691 | 0.520 | 11 |  |
| P - La | 0.059 | 0.840 | 326 | 0.905 | -0.500 | 0.584 | 14 |  |
| P - Nd | 0.088 | 0.721 | 307 | 0.824 | -0.394 | 0.532 | 19 |  |
| P - Y | -0.454 | 0.089 | 144 | 0.218 | -0.790 | 0.093 | 15 |  |
| S - Si | -0.281 | 0.174 | 186 | 0.328 | -0.616 | 0.141 | 25 |  |
| S - Ag | 0.162 | 0.450 | 258 | 0.613 | -0.270 | 0.540 | 24 |  |
| S - Al | -0.166 | 0.427 | 254 | 0.591 | -0.536 | 0.257 | 25 |  |
| S - Cd | 0.314 | 0.220 | 202 | 0.382 | -0.211 | 0.698 | 17 |  |
| S - Co | 0.023 | 0.920 | 340 | 0.950 | -0.424 | 0.461 | 21 |  |
| S - Cu | -0.272 | 0.189 | 190 | 0.349 | -0.610 | 0.151 | 25 |  |
| S - Fe | -0.022 | 0.916 | 339 | 0.948 | -0.424 | 0.387 | 25 |  |
| S - Mn | -0.196 | 0.347 | 233 | 0.523 | -0.557 | 0.227 | 25 |  |
| S - Ni | -0.287 | 0.264 | 220 | 0.422 | -0.683 | 0.240 | 17 |  |
| S - Pb | -0.090 | 0.669 | 296 | 0.793 | -0.478 | 0.327 | 25 |  |
| S - Sb | 0.286 | 0.235 | 206 | 0.401 | -0.207 | 0.663 | 19 |  |
| S - Zn | -0.172 | 0.410 | 249 | 0.578 | -0.540 | 0.251 | 25 |  |
| S - Ce | 0.011 | 0.964 | 346 | 0.978 | -0.470 | 0.487 | 18 |  |
| S - Dy | 0.132 | 0.613 | 284 | 0.757 | -0.385 | 0.587 | 17 |  |
| S - Gd | -0.231 | 0.427 | 255 | 0.588 | -0.688 | 0.357 | 14 |  |
| S - La | 0.096 | 0.715 | 304 | 0.826 | -0.416 | 0.562 | 17 |  |
| S - Nd | -0.346 | 0.098 | 151 | 0.227 | -0.665 | 0.079 | 24 |  |
| S - Y | 0.183 | 0.468 | 261 | 0.630 | -0.324 | 0.608 | 18 |  |
| Si - Ag | -0.143 | 0.504 | 266 | 0.665 | -0.526 | 0.288 | 24 |  |
| Si - Al | 0.973 | 0.000 | 2 | 0.000 | 0.937 | 0.989 | 25 |  |
| Si - Cd | -0.113 | 0.667 | 293 | 0.799 | -0.573 | 0.402 | 17 |  |
| Si - Co | 0.656 | 0.001 | 74 | 0.006 | 0.300 | 0.851 | 21 |  |
| Si - Cu | 0.526 | 0.007 | 94 | 0.026 | 0.153 | 0.768 | 25 |  |
| Si - Fe | 0.207 | 0.321 | 231 | 0.488 | -0.217 | 0.565 | 25 |  |
| Si - Mn | 0.809 | 0.000 | 35 | 0.000 | 0.601 | 0.915 | 25 |  |
| Si - Ni | 0.561 | 0.019 | 111 | 0.060 | 0.095 | 0.826 | 17 |  |
| Si - Pb | 0.346 | 0.090 | 146 | 0.217 | -0.069 | 0.659 | 25 |  |
| Si - Sb | -0.374 | 0.115 | 164 | 0.246 | -0.715 | 0.111 | 19 |  |
| Si - Zn | 0.769 | 0.000 | 48 | 0.000 | 0.529 | 0.895 | 25 |  |
| Si - Ce | 0.280 | 0.261 | 218 | 0.420 | -0.230 | 0.669 | 18 |  |
| Si - Dy | 0.267 | 0.300 | 226 | 0.466 | -0.259 | 0.671 | 17 |  |
| Si - Gd | 0.266 | 0.358 | 235 | 0.535 | -0.324 | 0.707 | 14 |  |
| Si - La | 0.390 | 0.122 | 169 | 0.253 | -0.127 | 0.740 | 17 |  |
| Si - Nd | 0.442 | 0.031 | 119 | 0.091 | 0.034 | 0.723 | 24 |  |
| Si - Y | 0.181 | 0.473 | 264 | 0.629 | -0.326 | 0.607 | 18 |  |
| Ag - Al | -0.077 | 0.722 | 310 | 0.818 | -0.475 | 0.348 | 24 |  |
| Ag - Cd | -0.082 | 0.762 | 313 | 0.854 | -0.566 | 0.444 | 16 |  |
| Ag - Co | 0.326 | 0.160 | 181 | 0.311 | -0.150 | 0.679 | 20 |  |
| Ag - Cu | -0.277 | 0.191 | 191 | 0.351 | -0.620 | 0.155 | 24 |  |
| Ag - Fe | 0.037 | 0.862 | 333 | 0.909 | -0.382 | 0.444 | 24 |  |
| Ag - Mn | -0.063 | 0.771 | 314 | 0.862 | -0.464 | 0.361 | 24 |  |
| Ag - Ni | -0.021 | 0.940 | 343 | 0.962 | -0.523 | 0.492 | 16 |  |
| Ag - Pb | 0.186 | 0.384 | 245 | 0.550 | -0.247 | 0.557 | 24 |  |
| Ag - Sb | -0.121 | 0.633 | 287 | 0.774 | -0.567 | 0.380 | 18 |  |
| Ag - Zn | -0.279 | 0.187 | 189 | 0.346 | -0.621 | 0.152 | 24 |  |
| Ag - Ce | -0.507 | 0.038 | 124 | 0.107 | -0.800 | -0.020 | 17 |  |
| Ag - Dy | -0.306 | 0.249 | 215 | 0.407 | -0.704 | 0.239 | 16 |  |
| Ag - Gd | -0.055 | 0.859 | 332 | 0.908 | -0.600 | 0.525 | 13 |  |
| Ag - La | -0.394 | 0.131 | 171 | 0.269 | -0.751 | 0.142 | 16 |  |
| Ag - Nd | -0.555 | 0.006 | 92 | 0.023 | -0.792 | -0.173 | 23 |  |
| Ag - Y | -0.400 | 0.112 | 160 | 0.246 | -0.745 | 0.116 | 17 |  |
| Al - Cd | -0.174 | 0.504 | 267 | 0.663 | -0.614 | 0.348 | 17 |  |
| Al - Co | 0.625 | 0.002 | 81 | 0.011 | 0.251 | 0.836 | 21 |  |
| Al - Cu | 0.560 | 0.004 | 84 | 0.015 | 0.200 | 0.787 | 25 |  |
| Al - Fe | 0.302 | 0.142 | 175 | 0.285 | -0.118 | 0.631 | 25 |  |
| Al - Mn | 0.778 | 0.000 | 45 | 0.000 | 0.544 | 0.900 | 25 |  |
| Al - Ni | 0.456 | 0.066 | 133 | 0.174 | -0.047 | 0.774 | 17 |  |
| Al - Pb | 0.352 | 0.085 | 141 | 0.211 | -0.063 | 0.663 | 25 |  |
| Al - Sb | -0.328 | 0.170 | 185 | 0.323 | -0.689 | 0.162 | 19 |  |
| Al - Zn | 0.813 | 0.000 | 34 | 0.000 | 0.608 | 0.916 | 25 |  |
| Al - Ce | 0.288 | 0.247 | 212 | 0.408 | -0.221 | 0.674 | 18 |  |
| Al - Dy | 0.333 | 0.191 | 192 | 0.349 | -0.190 | 0.709 | 17 |  |
| Al - Gd | 0.226 | 0.436 | 257 | 0.596 | -0.361 | 0.685 | 14 |  |
| Al - La | 0.397 | 0.115 | 163 | 0.247 | -0.119 | 0.744 | 17 |  |
| Al - Nd | 0.437 | 0.033 | 122 | 0.095 | 0.028 | 0.720 | 24 |  |
| Al - Y | 0.168 | 0.505 | 268 | 0.661 | -0.337 | 0.599 | 18 |  |
| Cd - Co | 0.233 | 0.368 | 239 | 0.541 | -0.293 | 0.651 | 17 |  |
| Cd - Cu | -0.049 | 0.852 | 330 | 0.906 | -0.529 | 0.454 | 17 |  |
| Cd - Fe | 0.061 | 0.815 | 322 | 0.889 | -0.445 | 0.538 | 17 |  |
| Cd - Mn | -0.145 | 0.580 | 279 | 0.729 | -0.595 | 0.375 | 17 |  |
| Cd - Ni | 0.448 | 0.145 | 177 | 0.287 | -0.189 | 0.819 | 12 |  |
| Cd - Pb | 0.588 | 0.013 | 103 | 0.044 | 0.135 | 0.838 | 17 |  |
| Cd - Sb | 0.400 | 0.140 | 173 | 0.283 | -0.158 | 0.764 | 15 |  |
| Cd - Zn | -0.135 | 0.606 | 282 | 0.754 | -0.588 | 0.383 | 17 |  |
| Cd - Ce | 0.084 | 0.795 | 319 | 0.875 | -0.529 | 0.639 | 12 |  |
| Cd - Dy | -0.014 | 0.966 | 347 | 0.977 | -0.596 | 0.577 | 12 |  |
| Cd - Gd | -0.321 | 0.365 | 236 | 0.544 | -0.799 | 0.405 | 10 |  |
| Cd - La | -0.014 | 0.966 | 347 | 0.977 | -0.596 | 0.577 | 12 |  |
| Cd - Nd | -0.441 | 0.076 | 135 | 0.198 | -0.767 | 0.066 | 17 |  |
| Cd - Y | 0.203 | 0.527 | 271 | 0.683 | -0.436 | 0.706 | 12 |  |
| Co - Cu | 0.084 | 0.716 | 305 | 0.824 | -0.372 | 0.508 | 21 |  |
| Co - Fe | -0.084 | 0.716 | 305 | 0.824 | -0.508 | 0.372 | 21 |  |
| Co - Mn | 0.635 | 0.002 | 78 | 0.009 | 0.268 | 0.841 | 21 |  |
| Co - Ni | 0.238 | 0.374 | 241 | 0.545 | -0.307 | 0.665 | 16 |  |
| Co - Pb | 0.338 | 0.134 | 172 | 0.274 | -0.124 | 0.679 | 21 |  |
| Co - Sb | -0.399 | 0.101 | 155 | 0.228 | -0.737 | 0.098 | 18 |  |
| Co - Zn | 0.151 | 0.515 | 270 | 0.669 | -0.313 | 0.556 | 21 |  |
| Co - Ce | 0.138 | 0.610 | 283 | 0.756 | -0.397 | 0.604 | 16 |  |
| Co - Dy | 0.121 | 0.656 | 292 | 0.789 | -0.412 | 0.592 | 16 |  |
| Co - Gd | 0.220 | 0.471 | 263 | 0.628 | -0.392 | 0.697 | 13 |  |
| Co - La | 0.126 | 0.641 | 290 | 0.775 | -0.407 | 0.596 | 16 |  |
| Co - Nd | 0.091 | 0.695 | 301 | 0.811 | -0.367 | 0.513 | 21 |  |
| Co - Y | 0.221 | 0.412 | 250 | 0.578 | -0.323 | 0.655 | 16 |  |
| Cu - Fe | 0.592 | 0.002 | 76 | 0.009 | 0.245 | 0.804 | 25 |  |
| Cu - Mn | 0.350 | 0.086 | 142 | 0.213 | -0.065 | 0.662 | 25 |  |
| Cu - Ni | 0.581 | 0.014 | 106 | 0.048 | 0.124 | 0.835 | 17 |  |
| Cu - Pb | 0.219 | 0.292 | 225 | 0.456 | -0.204 | 0.574 | 25 |  |
| Cu - Sb | 0.318 | 0.185 | 188 | 0.346 | -0.174 | 0.682 | 19 |  |
| Cu - Zn | 0.797 | 0.000 | 40 | 0.000 | 0.578 | 0.909 | 25 |  |
| Cu - Ce | 0.030 | 0.906 | 338 | 0.941 | -0.455 | 0.501 | 18 |  |
| Cu - Dy | 0.228 | 0.379 | 242 | 0.550 | -0.298 | 0.648 | 17 |  |
| Cu - Gd | -0.108 | 0.714 | 302 | 0.830 | -0.615 | 0.462 | 14 |  |
| Cu - La | 0.230 | 0.374 | 240 | 0.546 | -0.296 | 0.649 | 17 |  |
| Cu - Nd | 0.317 | 0.131 | 170 | 0.270 | -0.111 | 0.646 | 24 |  |
| Cu - Y | -0.131 | 0.604 | 281 | 0.755 | -0.574 | 0.371 | 18 |  |
| Fe - Mn | 0.168 | 0.421 | 252 | 0.586 | -0.254 | 0.537 | 25 |  |
| Fe - Ni | 0.252 | 0.328 | 232 | 0.497 | -0.274 | 0.663 | 17 |  |
| Fe - Pb | 0.128 | 0.543 | 274 | 0.696 | -0.293 | 0.507 | 25 |  |
| Fe - Sb | 0.572 | 0.011 | 101 | 0.037 | 0.145 | 0.819 | 19 |  |
| Fe - Zn | 0.493 | 0.012 | 102 | 0.042 | 0.109 | 0.749 | 25 |  |
| Fe - Ce | -0.387 | 0.113 | 161 | 0.246 | -0.730 | 0.112 | 18 |  |
| Fe - Dy | -0.113 | 0.667 | 293 | 0.799 | -0.573 | 0.402 | 17 |  |
| Fe - Gd | -0.433 | 0.122 | 168 | 0.255 | -0.790 | 0.144 | 14 |  |
| Fe - La | -0.355 | 0.162 | 182 | 0.312 | -0.722 | 0.166 | 17 |  |
| Fe - Nd | -0.057 | 0.790 | 318 | 0.872 | -0.460 | 0.365 | 24 |  |
| Fe - Y | -0.397 | 0.103 | 156 | 0.231 | -0.736 | 0.100 | 18 |  |
| Mn - Ni | 0.321 | 0.209 | 196 | 0.374 | -0.204 | 0.702 | 17 |  |
| Mn - Pb | 0.265 | 0.200 | 195 | 0.360 | -0.157 | 0.606 | 25 |  |
| Mn - Sb | -0.619 | 0.005 | 87 | 0.019 | -0.842 | -0.216 | 19 |  |
| Mn - Zn | 0.583 | 0.002 | 79 | 0.010 | 0.233 | 0.800 | 25 |  |
| Mn - Ce | -0.102 | 0.687 | 298 | 0.809 | -0.554 | 0.396 | 18 |  |
| Mn - Dy | 0.123 | 0.639 | 289 | 0.777 | -0.394 | 0.580 | 17 |  |
| Mn - Gd | 0.059 | 0.840 | 326 | 0.905 | -0.500 | 0.584 | 14 |  |
| Mn - La | 0.086 | 0.743 | 311 | 0.839 | -0.425 | 0.555 | 17 |  |
| Mn - Nd | 0.253 | 0.233 | 205 | 0.399 | -0.180 | 0.604 | 24 |  |
| Mn - Y | 0.049 | 0.848 | 329 | 0.905 | -0.440 | 0.515 | 18 |  |
| Ni - Pb | 0.294 | 0.252 | 216 | 0.409 | -0.232 | 0.687 | 17 |  |
| Ni - Sb | 0.442 | 0.114 | 162 | 0.247 | -0.133 | 0.794 | 14 |  |
| Ni - Zn | 0.370 | 0.144 | 176 | 0.287 | -0.150 | 0.730 | 17 |  |
| Ni - Ce | 0.125 | 0.670 | 297 | 0.791 | -0.448 | 0.626 | 14 |  |
| Ni - Dy | 0.002 | 0.994 | 351 | 0.994 | -0.541 | 0.545 | 14 |  |
| Ni - Gd | -0.382 | 0.247 | 211 | 0.410 | -0.806 | 0.302 | 11 |  |
| Ni - La | 0.059 | 0.840 | 326 | 0.905 | -0.500 | 0.584 | 14 |  |
| Ni - Nd | 0.050 | 0.854 | 331 | 0.906 | -0.470 | 0.544 | 16 |  |
| Ni - Y | -0.024 | 0.935 | 342 | 0.959 | -0.560 | 0.526 | 14 |  |
| Pb - Sb | -0.137 | 0.576 | 278 | 0.728 | -0.566 | 0.351 | 19 |  |
| Pb - Zn | 0.352 | 0.084 | 140 | 0.211 | -0.062 | 0.663 | 25 |  |
| Pb - Ce | -0.032 | 0.900 | 337 | 0.937 | -0.503 | 0.453 | 18 |  |
| Pb - Dy | 0.093 | 0.722 | 308 | 0.823 | -0.419 | 0.560 | 17 |  |
| Pb - Gd | 0.051 | 0.864 | 334 | 0.908 | -0.506 | 0.578 | 14 |  |
| Pb - La | 0.093 | 0.722 | 308 | 0.823 | -0.419 | 0.560 | 17 |  |
| Pb - Nd | -0.117 | 0.588 | 280 | 0.737 | -0.506 | 0.312 | 24 |  |
| Pb - Y | 0.036 | 0.887 | 335 | 0.929 | -0.450 | 0.506 | 18 |  |
| Sb - Zn | -0.018 | 0.943 | 344 | 0.962 | -0.479 | 0.452 | 19 |  |
| Sb - Ce | 0.068 | 0.817 | 324 | 0.885 | -0.493 | 0.589 | 14 |  |
| Sb - Dy | -0.108 | 0.714 | 302 | 0.830 | -0.615 | 0.462 | 14 |  |
| Sb - Gd | -0.655 | 0.029 | 118 | 0.086 | -0.905 | -0.070 | 11 |  |
| Sb - La | -0.073 | 0.805 | 321 | 0.881 | -0.592 | 0.490 | 14 |  |
| Sb - Nd | -0.220 | 0.381 | 243 | 0.550 | -0.632 | 0.289 | 18 |  |
| Sb - Y | -0.147 | 0.615 | 285 | 0.758 | -0.639 | 0.430 | 14 |  |
| Zn - Ce | 0.059 | 0.817 | 323 | 0.887 | -0.432 | 0.523 | 18 |  |
| Zn - Dy | 0.223 | 0.390 | 246 | 0.556 | -0.303 | 0.645 | 17 |  |
| Zn - Gd | -0.138 | 0.637 | 288 | 0.776 | -0.634 | 0.437 | 14 |  |
| Zn - La | 0.297 | 0.248 | 213 | 0.408 | -0.229 | 0.688 | 17 |  |
| Zn - Nd | 0.423 | 0.039 | 125 | 0.110 | 0.012 | 0.713 | 24 |  |
| Zn - Y | -0.123 | 0.627 | 286 | 0.770 | -0.568 | 0.378 | 18 |  |
| Ce - Dy | 0.863 | 0.000 | 50 | 0.000 | 0.644 | 0.951 | 17 |  |
| Ce - Gd | 0.916 | 0.000 | 43 | 0.000 | 0.743 | 0.975 | 14 |  |
| Ce - La | 0.971 | 0.000 | 6 | 0.000 | 0.916 | 0.990 | 17 |  |
| Ce - Nd | 0.955 | 0.000 | 11 | 0.000 | 0.876 | 0.984 | 18 |  |
| Ce - Y | 0.953 | 0.000 | 15 | 0.000 | 0.869 | 0.984 | 17 |  |
| Dy - Gd | 0.859 | 0.000 | 61 | 0.000 | 0.593 | 0.956 | 14 |  |
| Dy - La | 0.877 | 0.000 | 42 | 0.000 | 0.678 | 0.957 | 17 |  |
| Dy - Nd | 0.926 | 0.000 | 26 | 0.000 | 0.798 | 0.974 | 17 |  |
| Dy - Y | 0.918 | 0.000 | 32 | 0.000 | 0.768 | 0.972 | 16 |  |
| Gd - La | 0.886 | 0.000 | 57 | 0.000 | 0.660 | 0.965 | 14 |  |
| Gd - Nd | 0.938 | 0.000 | 33 | 0.000 | 0.806 | 0.981 | 14 |  |
| Gd - Y | 0.973 | 0.000 | 22 | 0.000 | 0.905 | 0.992 | 13 |  |
| La - Nd | 0.968 | 0.000 | 8 | 0.000 | 0.909 | 0.989 | 17 |  |
| La - Y | 0.932 | 0.000 | 28 | 0.000 | 0.806 | 0.977 | 16 |  |
| Nd - Y | 0.775 | 0.000 | 64 | 0.001 | 0.471 | 0.914 | 18 |  |
| a. Estimation is based on Fisher's r-to-z transformation. | | | | | | |  |  |
| b. Estimation of standard error is based on the formula proposed by Fieller, Hartley, and Pearson. | | | | | | |  |  |
|  |  |  |  |  |  |  |  |  |
