# Supplementary figures and images for: Geochemical signatures in plastic debris from the Curonian Lagoon, Lithuania
Source: PLoS One. 2026 Feb 2;21(2):e0340582. doi: 10.1371/journal.pone.0340582 (PMC12863676; doi:10.1371/journal.pone.0340582)

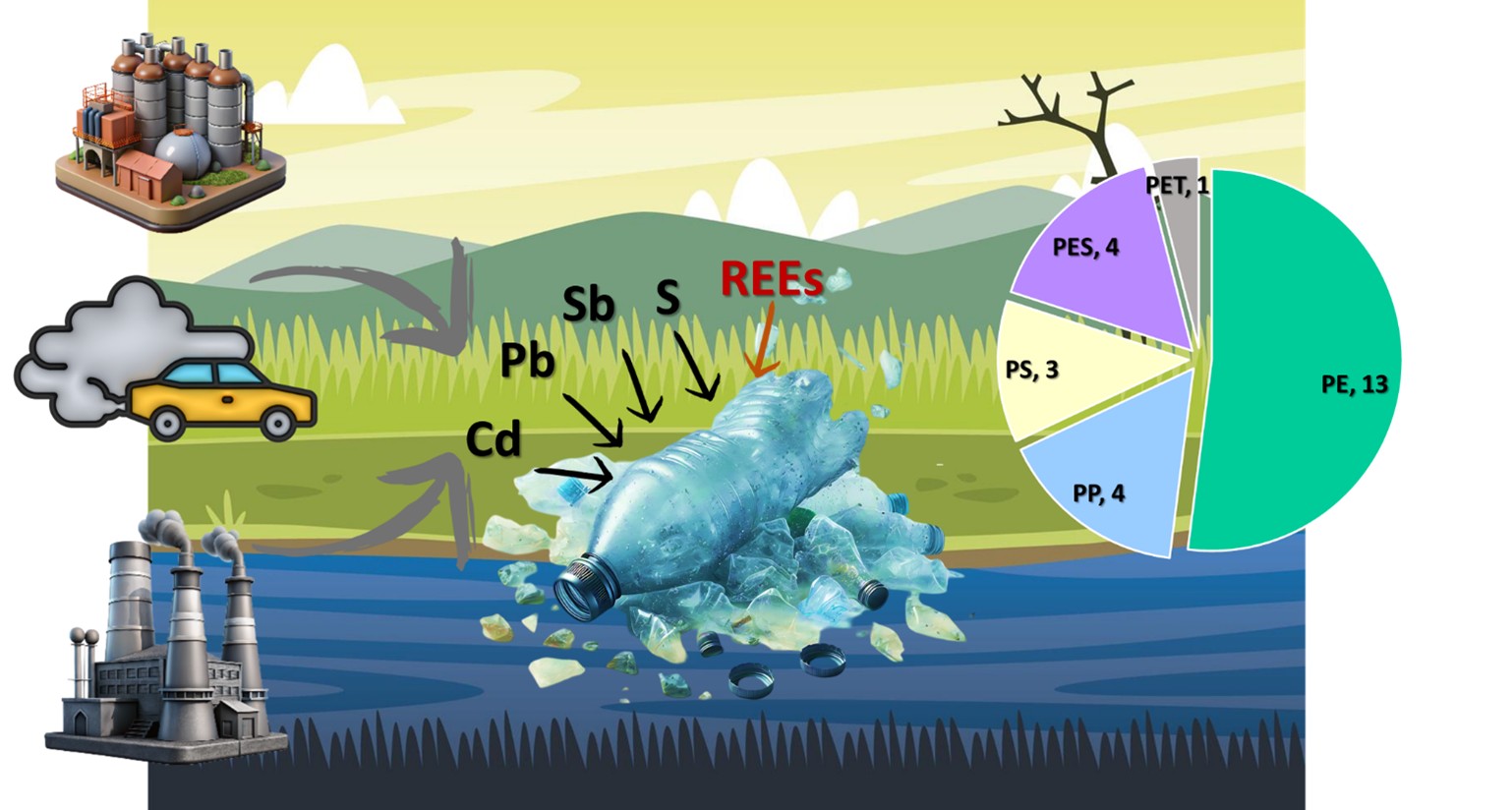

Supplement: S1 Fig — (JPG) [file pone.0340582.s003.jpg]
